# Glycosylation generates an efficacious and immunogenic vaccine against H7N9 influenza virus

**DOI:** 10.1371/journal.pbio.3001024

**Published:** 2020-12-23

**Authors:** Jin Il Kim, Sehee Park, Joon-Yong Bae, Sunmi Lee, Jeonghun Kim, Gayeong Kim, Kirim Yoo, Jun Heo, Yong Seok Kim, Jae Soo Shin, Mee Sook Park, Man-Seong Park

**Affiliations:** 1 Department of Microbiology, Institute for Viral Diseases, Korea University College of Medicine, Seoul, Republic of Korea; 2 Biosafety Center, Korea University College of Medicine, Seoul, Republic of Korea; 3 Il Yang Pharmaceutical Co., Yongin, Gyeonggi-do, Republic of Korea; Weatherall Institute of Molecular Medicine, UNITED KINGDOM

## Abstract

Zoonotic avian influenza viruses pose severe health threats to humans. Of several viral subtypes reported, the low pathogenic avian influenza H7N9 virus has since February 2013 caused more than 1,500 cases of human infection with an almost 40% case-fatality rate. Vaccination of poultry appears to reduce human infections. However, the emergence of highly pathogenic strains has increased concerns about H7N9 pandemics. To develop an efficacious H7N9 human vaccine, we designed vaccine viruses by changing the patterns of *N*-linked glycosylation (NLG) on the viral hemagglutinin (HA) protein based on evolutionary patterns of H7 HA NLG changes. Notably, a virus in which 2 NLG modifications were added to HA showed higher growth rates in cell culture and elicited more cross-reactive antibodies than did other vaccine viruses with no change in the viral antigenicity. Developed into an inactivated vaccine formulation, the vaccine virus with 2 HA NLG additions exhibited much better protective efficacy against lethal viral challenge in mice than did a vaccine candidate with wild-type (WT) HA by reducing viral replication in the lungs. In a ferret model, the 2 NLG-added vaccine viruses also induced hemagglutination-inhibiting antibodies and significantly suppressed viral replication in the upper and lower respiratory tracts compared with the WT HA vaccines. In a mode of action study, the HA NLG modification appeared to increase HA protein contents incorporated into viral particles, which would be successfully translated to improve vaccine efficacy. These results suggest the strong potential of HA NLG modifications in designing avian influenza vaccines.

## Abstract 2

This study shows that changing the pattern of N-glycosylation of the pathogenic avian influenza H7N9 virus hemagglutinin protein increases the amount of hemagglutinin incorporated into the viral membrane; the candidate vaccine virus induces neutralizing antibodies and protects animal models from lethal viral challenge.

## Introduction

Influenza A virus (IAV), a member of the family *Orthomyxoviridae*, infects various animal hosts, including humans [1]; however, the transfer of the virus between different host species is typically restricted by transmission barriers. For example, the hemagglutinin (HA) protein, which is located on the surface of the influenza virus, binds to sialic acid (SA)-containing receptors on the host cell membrane to mediate cell entry. Given the different receptor-binding specificities of the HAs of avian and human influenza viruses, avian IAVs are unlikely to invade the human respiratory tract because of the preference of their HA for α2,3-SA-containing receptors rather than α2,6-SA-containing receptors, which the HA of human IAVs preferentially binds [2,3]. To overcome this species barrier, the avian virus may need to undergo a prior period of adaptation in a different host or to obtain certain genetic mutation(s) or reassortment that enables sustained transmission within a new host species [4,5]. In this regard, low pathogenic avian influenza A (H7N9) virus strains may be a good example of both adaptation and genetic reassortment [6,7]. Before or during the transition from avian to human hosts in 2013, the genome arrangement of the H7N9 strain appears to have been largely the result of multiple events of adaptation and genetic exchange in different animal hosts [8]. However, after acquiring a certain level of fitness, the H7N9 virus has evolved into a zoonotic pathogen that has posed a pandemic threat [9]. During the 2012 to 2017 influenza seasons, the H7N9 virus extended its influence into many different geographical regions in China and caused more than 540 human infection cases, which comprised more than one-third of the total laboratory-confirmed human cases with the virus [7,9]. As of December 4, 2019, of the 1,568 confirmed human cases of avian influenza H7N9 infection, 616 had resulted in death (case-fatality rate = 39.29%) [9]. Although the number of human infection cases has been reduced, probably due to the massive vaccination of poultry animals in China [10], it should be noted that highly pathogenic avian influenza (HPAI) H7N9 strains have emerged in recent years [9]. As demonstrated in the global dissemination of HPAI H5Nx viruses [11], reassortment events in HPAI H7N9 strains may generate novel viruses that pose significant public health threats to humans [12].

To minimize the threat of a potential H7N9 pandemic, a vaccine that protects against infection by the virus is considered to be the first choice [13]. Several H7N9 vaccine candidates have been tested in clinical trials in humans in the presence or absence of an adjuvant [14–16]. Unfortunately, however, the immunogenic properties of the stockpiled current H7N9 candidate vaccine viruses have been less than promising to date [14–16]: None of the candidates induced an antibody titer response of more than 40 hemagglutination inhibition (HI) units, which is considered to be the minimum level of protective antibody titer elicited after vaccination [17,18], and the seroconversion rate in individuals who were subjected to a prime-boost vaccination strategy even with adjuvant supplementation was only approximately 60% [14,19]. Only in the recently reported study, it was revealed that high antigen dose formulation with adjuvant supplementation might result in higher immune responses [20]. A delayed immune response and the low avidity of H7N9-induced antibodies have been suggested as the reasons for the low immunogenicity of these vaccines [21]. In other vaccine trials against the H7N1 and H7N7 avian IAVs, the vaccine candidates also appeared to be less immunogenic [22,23]. Hence, it is imperative to design an effective, highly immunogenic H7N9 vaccine to address the current H7N9 vaccine issues before the virus spreads much further.

N-linked glycosylation (NLG) is an essential posttranslational modification that occurs on the HA and neuraminidase (NA) surface glycoproteins of influenza viruses harboring a glycan on the asparagine (Asn, N) residue when the amino acid sequence consists of “N-X-S/T” (where “X” is any amino acid except proline) [24]. Changes in the NLG status of the HA head region of influenza viruses may affect virus binding to SA-containing receptors and eventually transform viral antigenicity [25]. Additionally, changes in NLG status often result in the masking of certain antigenic sites and can constitute an immune evasion mechanism by which a virus hides from recognition by host immune machineries [26]. Previous studies suggest that the introduction of NLG modifications to the HA head region can increase the immunogenicity of IAV. For example, the introduction of 2 additional NLG sites in the head region of the HA of a pandemic (H1N1) 2009 virus was shown to induce broad-spectrum antibody responses [27,28], whereas 2 other studies demonstrated different effects of NLG modifications on the immunogenicity of H5N1 vaccines [29,30]. For the H7-subtype viruses, the presence or absence of NLG at HA amino acid residue 133 seems to determine the ability of the virus to evade recognition by neutralizing antibodies [31]. Therefore, the effects of NLG modification on the generation of an innate immune response and its subsequent benefit to humoral immunity must be balanced with its effects on viral infectivity when developing candidate vaccine strains.

Here, we investigated the NLG patterns of the HAs of various H7-subtype viruses and used an NLG modification strategy to generate efficacious H7N9 candidate vaccines. On the basis of the HA NLG patterns identified, we generated mutant viruses and observed their enhanced replication properties in cells and immunogenicity in vivo. In an inactivated vaccine formulation, one of the HA NLG-mutant vaccine viruses exhibited higher effectiveness in mice and ferrets than an H7N9 vaccine, and we investigated a mode of action of the HA NLG modification strategy.

## Results

### NLG patterns on hemagglutinin and the generation of NLG-mutant H7 viruses

To determine the changing patterns of NLG modifications in the H7 HA proteins, we obtained the HA genetic sequences of H7N1, H7N2, H7N3, H7N7, and H7N9 subtype viruses from the National Center for Biotechnology Information (NCBI) database. From the 1,361 HA sequences, we identified 7 common potential NLG sites at HA1 amino acid residues 22, 38, 133, 158, and 240, and at HA2 amino acid residues 82 and 154 (H3 numbering) based on the NLG sequons [28] (S1 Table). Of the 3 glycosites at residues 133, 158, and 240 in the HA globular head region (S1 Fig), glycosites 133 and 158 were used by less than 8% of the unique H7 HA sequences, and that percentage was even lower in the HA from the H7N9 subtype (S1 Table). To analyze the effects of the NLG modifications in the HA globular head region on viral replication and immunogenicity, we generated four 7:1 NLG-mutant viruses using the HA of the influenza A/Anhui/1/2013 (H7N9 subtype) virus with the genetic backbone from the influenza A/Korea/01/2009 (pandemic (H1N1) 2009 strain) virus by plasmid-based reverse genetics [28]. We made 2 single mutants in which an NLG modification was added at either residue 133 (hereafter termed rH7+133) or residue 158 (rH7+158), 1 mutant in which NLG modifications were added at both residues 133 and 158 (rH7+133+158), and 1 mutant in which an NLG modification was removed from residue 240 (rH7-240). The sequence encoding wild-type (WT) HA from influenza A/Anhui/1/2013 was used to generate a control parental HA-reassortant virus (rH7) (S2 Table).

**Fig 1:**
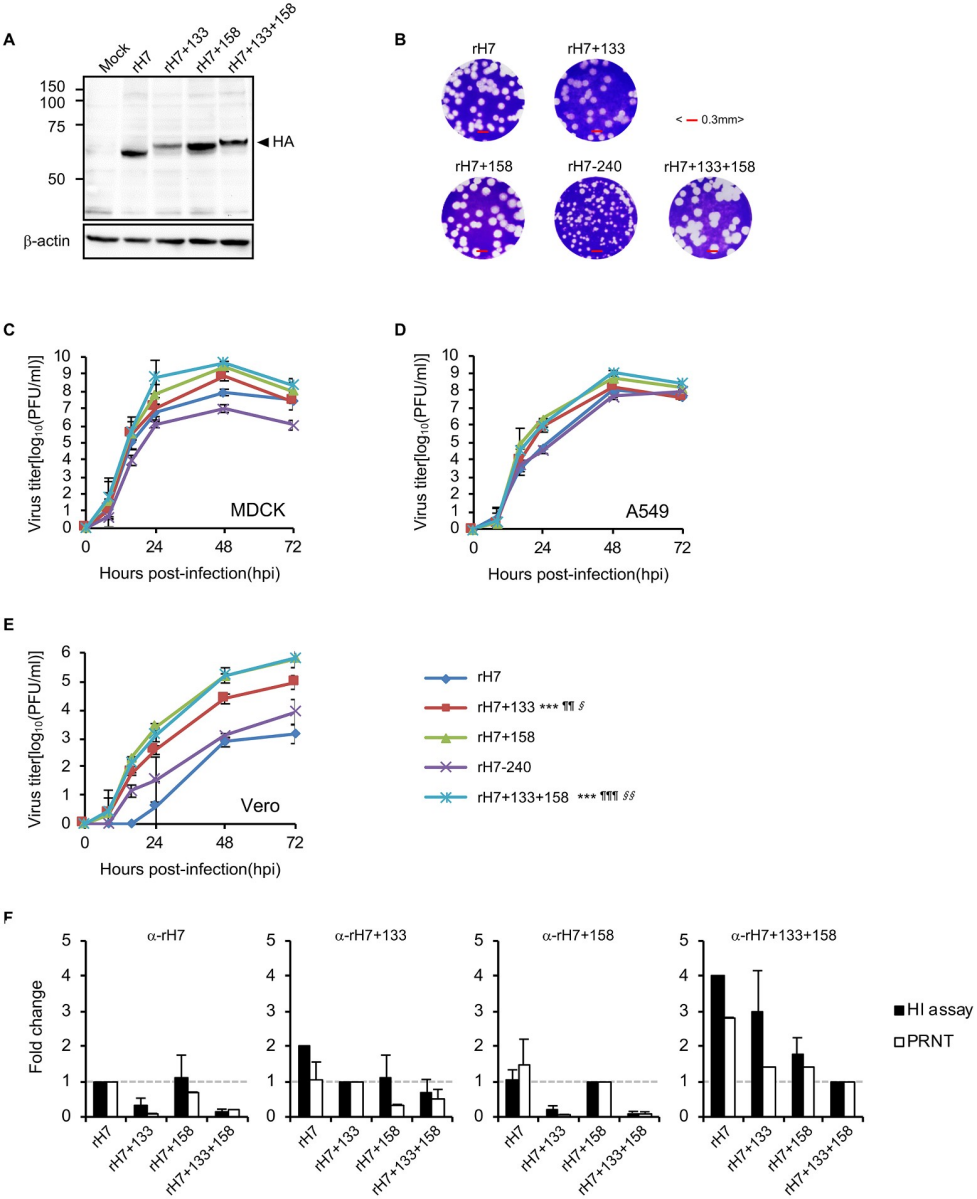
Effects of HA NLG modifications on viral characteristics. (**A**) Western blot analysis was performed using MDCK cell lysates infected with the indicated viruses or treated with PBS (Mock). β-Actin was used as a loading control. (**B**) Plaque phenotypes of the indicated NLG-mutant viruses were observed in MDCK cells (scale bars, 0.3 mm). Growth kinetics of the 4 NLG-mutant viruses in MDCK (**C**), A549 (**D**), or Vero (**E**) cells. The data represent the mean ± SD (denoted with error bars) of 3 independent experiments. The titers of the rH7 virus were used as controls for the assessment of statistical significance of the results. *P* values (§, *P* < 0.05; §§ and ¶¶, *P* < 0.01; and ¶¶¶ and ***, *P* < 0.001) were determined by 1-way ANOVA and confirmed by Tukey multiple comparison test. (**F**) Serological responses of antisera from guinea pigs that had been intranasally infected with NLG-mutant viruses (rH7, rH7+133, rH7+158, or rH7+133+158) were determined using HI (black bars) and PRNT (white bars) assays. The data are presented as the GMT ± SD of the fold changes, based on those of each homologous titer between a virus and its antiserum (e.g., fold changes of HI titers of rH7+133, rH7+158, or rH7+133+158 were calculated based on the HI titer of a homologous reaction between rH7 and its antiserum), from 4 (HI assay) and 2 (PRNT) independent experiments. Serum of naïve guinea pigs, used as a control, resulted in no HI (PRNT) reaction. Please see S1 Data for the numerical values used in Fig 1. ANOVA, analysis of variance; GMT, geometric mean titer; HA, hemagglutinin; HI, hemagglutination inhibition; MDCK, Madin-Darby canine kidney; NLG, N-linked glycosylation; PBS, phosphate buffered saline; PRNT, plaque-reduction neutralization test; SD, standard deviation.

To confirm that the mutant viruses exhibited the expected changes in NLG status, we infected Madin-Darby canine kidney (MDCK) cells with each virus and analyzed the mobility shifts of the HA proteins expressed in the infected cells. The HAs in cells that were infected with the rH7+133 and rH7+158 viruses migrated slowly compared to the HAs in cells that were infected with the parental rH7 virus (Fig 1A and S2 Fig), which might indicate the addition of an NLG modification at each of the designated residues. Consistent with this result, we observed that the HA from the rH7+133+158 virus, which should contain 2 additional NLG modifications, had the slowest mobility. However, we could not detect the HA of the rH7-240 virus in the same western blot analysis (S2 Fig).

### Replication properties of the NLG-mutant viruses in mammalian cells

A high growth rate is one of the critical issues of candidate vaccine viruses. Prior to the replication property analysis of NLG-mutant viruses in the cell lines, we examined the plaque sizes of the NLG-mutant viruses in MDCK cells. The rH7+133, rH7+158, and rH7+133+158 viruses produced similarly sized or larger plaques than those produced by the parental rH7 virus, whereas the rH7-240 virus produced extremely small plaques (Fig 1B). Notably, the increased plaque sizes of the viruses in which NLG modifications were introduced (rH7+133, rH7+158, and rH7+133+158) seemed to correlate with the ability of the viruses to replicate in MDCK, A549, and Vero cells (Fig 1C–1E). Among these 3 viruses, rH7+158 and rH7+133+158 showed higher replication rates than rH7+133 in all of the cells tested, and the rH7+133+158 virus showed the highest replication, especially in MDCK cells, compared to that of the parental rH7 virus. However, the rH7-240 virus, in which an NLG modification had been removed, was less competent than all of the other viruses in terms of its replication kinetics in the tested cells (Fig 1C–1E), which explains our observation of decreased plaque sizes of rH7-240 (Fig 1B). In a mouse model, the NLG-mutant viruses did not exhibit severe pathogenicity (S3 Fig and S3 Table), which can be interpreted as another important factor for the candidate vaccine viruses. Taken together, our results suggest that the viruses in which NLG modifications were introduced into HA exhibit high-yield replication properties in vitro and less adverse pathogenic effects in vivo.

### Immunogenic properties of the NLG-mutant viruses

To analyze the vaccine potential of the viruses with NLG-mutant HA, we tested the immunogenic properties of the viruses using an HI assay and a plaque-reduction neutralization test (PRNT) (Fig 1F and S4 Table). The rH7-240 virus was not included in the serological analysis because of its low hemagglutination assay titer. In the HI assay, the antiserum from guinea pigs that had been infected with either the rH7 or rH7+158 virus exhibited less competence against the viruses harboring the additional NLG modification at HA residue 133 (rH7+133 and rH7+133+158) (S4 Table). However, the antiserum from rH7+133-infected guinea pigs exhibited higher HI titers against rH7, rH7+158, and rH7+133+158 than the antisera from rH7- or rH7+158-infected guinea pigs. The broad HI reactiveness was found for the antiserum from the guinea pigs that had been infected with the rH7+133+158 virus. Relative to the homologous HI titer determined between the antiserum from rH7+133+158-infected guinea pigs and the rH7+133+158 virus, the HI titers of the antiserum from rH7+133+158-infected guinea pigs resulted in increases of more than 1.5-fold against all the tested viruses and up to a 4-fold increase against the parental rH7 virus (Fig 1F and S4 Table). As observed in the HI assay results, the PRNT titers of each antiserum were similarly determined for all of the viruses tested. Compared with the homologous PRNT titers, the antiserum from rH7+133+158-infected guinea pigs exhibited the highest increases in PRNT titers against all the tested viruses, including the rH7-240 virus (Fig 1F and S4 Table). In summary, our results demonstrate that the NLG modifications introduced at HA residues 133 and 158 may induce HI- and PRNT-reactive antibodies not only against the parental rH7 but also against other NLG variants.

### H7N9 candidate vaccine viruses with HA NLG modifications

**Fig 2:**
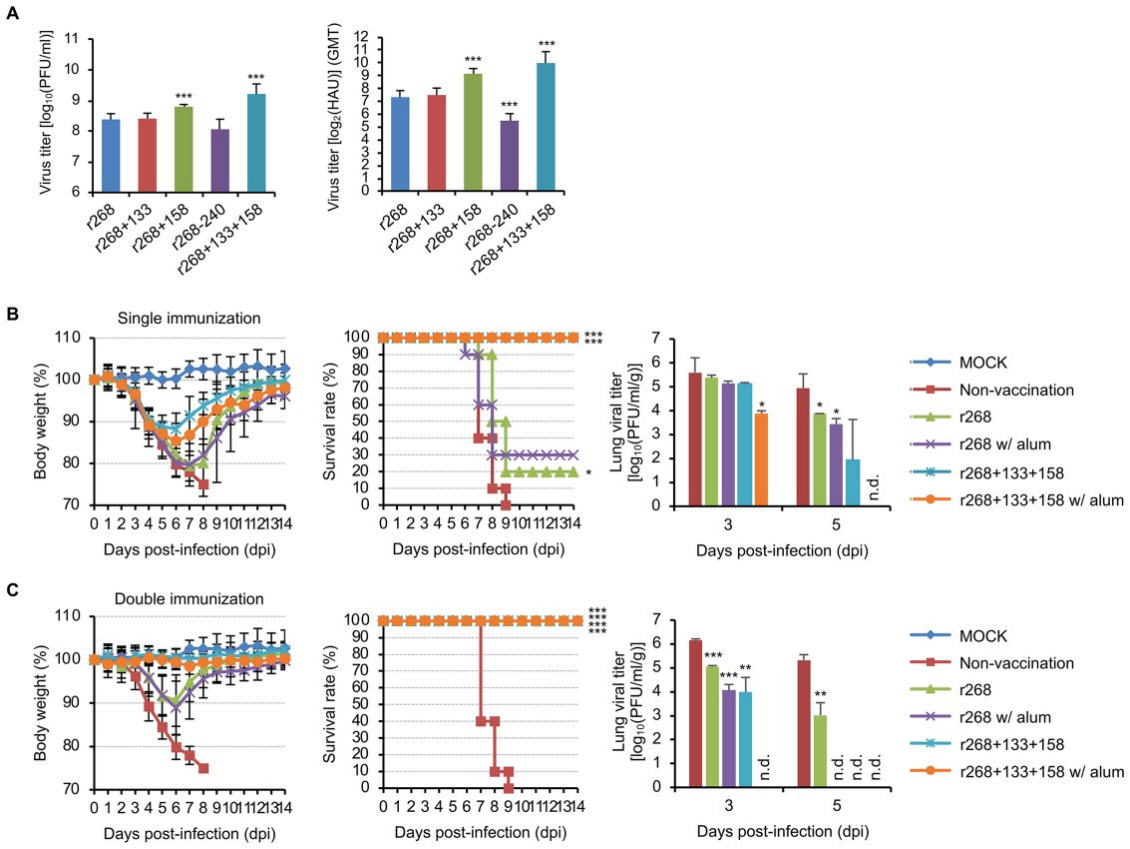
Protective efficacy of the candidate vaccines in mice. The growth rates and HA contents (**A**) of the candidate vaccine viruses with NLG-modified HA (r268+133, r268+158, r268-240, and r268+133+158) or of r268 were analyzed after collecting the allantoic fluids from chicken eggs at 48 hours after inoculation with 10^3^ PFU of each virus. The allantoic fluids were then used in a plaque assay, using MDCK cells, or in an HA assay. The data are the mean ± SD of 3 independent experiments. The statistical significance of the differences in viral titers relative to those in the allantoic fluids from r268-injected eggs was assessed by Student *t* test (***, *P* < 0.001). Body weight changes and survival rates of the PBS-injected (nonvaccinated) and vaccinated mice and viral titers in their lungs were analyzed for 14 days after lethal viral challenge (**B**, single immunization and **C**, double immunization). The mice in the mock group were vaccinated and challenged with PBS. The statistical significance of the differences in viral titers relative to those in the nonvaccinated mice was assessed by Student *t* test (*, *P* < 0.05; **, *P* < 0.01; and ***, *P* < 0.001). Please see S2 Data for the numerical values used in Fig 2. HA, hemagglutinin; MDCK, Madin-Darby canine kidney; n.d., not detected; NLG, N-linked glycosylation; PFU, plaque-forming unit.

To determine the feasibility of the virus expressing the double-NLG-mutant HA to develop an efficacious H7N9 vaccine, we generated an H7N9 vaccine virus strain (r268+133+158), which encodes the HA with 2 additional NLG sites and the original NA of influenza A/Anhui/1/2013 (H7N9) on the backbone of the A/Puerto Rico/8/1934 (PR8) human vaccine strain of IAVs. We also generated vaccine viruses with a single NLG modification either added (r268+133 and r268+158) or removed (r268-240) for comparison. Similar to one of the developed H7N9 candidate vaccine viruses [32], we also generated r268 that expresses the WT HA and NA of influenza A/Anhui/1/2013 for use as a control vaccine virus. In chick embryos, r268+133+158 showed the highest growth rate of any of the viruses tested at 48 hours post-infection (hpi). Consistent with this observation, the HA titer of r268+133+158 exceeded that of any of the other vaccine viruses at the same time point (Fig 2A).

### Protective efficacy of the vaccine harboring 2 additional HA NLG modifications in mice

Based on its highest replication properties and HA contents in the eggs (Fig 2A), r268+133+158 was selected for the assessment of its protective efficacy in mice. First, in the HI assay using the antisera collected from the single- or double-vaccinated mice, r268 elicited HI-reactive antibodies only in mice that were vaccinated with a double immunization (priming and boosting) strategy. In contrast, a single immunization of mice with r268+133+158 induced HI titers of 20.00 to 36.23 and 13.46 to 24.38 in the presence and absence of adjuvant, respectively (Table 1). When the mice were boosted with a second injection of r268+133+158, the mouse antisera exhibited 2- or 3-fold higher HI titers (63.50 to 97.52), and adjuvant supplementation resulted in much higher HI titers (211.1 to 262.5) (Table 1). As expected from the HI assay results, viral replication in the lungs of immunized mice was less efficient in r268+133+158-vaccinated mice than in r268-vaccinated mice. Mice that had been subjected to a double immunization strategy with r268+133+158 in the presence of adjuvant showed no signs of viral replication in their lungs, even from 3 days after the viral infection. In contrast, mice that had been immunized in a similar manner with r268 exhibited no viral replication in their lungs only in the case of double immunization with the presence of adjuvant (Fig 2B and 2C).

**Table 1:** Immunogenicity of single- or double-immunized mouse antiserum.

Virus	Geometric mean HI-reactive antibody titers of immunized mouse antiserum (95% CI)^†^							
	Single immunization				Double immunization			
	α-r268	α-r268 w/ alum adjuvant	α-r268+133+158	α-r268+133+158 w/ alum adjuvant	α-r268	α-r268 w/ alum adjuvant	α-r268+133+158	α-r268+133+158 w/ alum adjuvant
r268	0	0	24.38 (17.83–33.33)	36.23 (20.35–64.5)	29.72 (17.95–49.22)	56.57 (37.98–84.26)	97.52 (53–179.3)	262.5 (161.7–426.2)
r267	0	0	13.46 (9.55–18.96)	20 (13.81–28.96)	22.08 (12.4–39.31)	50.4 (27.82–91.28)	97.52 (53–179.4)	262.5 (161.7–426.2)
r268+133+158	0	0	24.28 (16.46–32.3)	35.64 (20.61–61.62)	12.6 (4.66–34.05)	25.2 (6.324–68.1)	63.5 (26.31–153.2)	211.1 (131.8–338.2)

^†^CI, confidence interval. Serum of naïve mice, used as a negative control, resulted in no HI reaction.

Against lethal viral challenge in mice, the r268+133+158 vaccine also exhibited much better efficacy than r268. Upon infection with the 5 MLD_50_ (50% mouse lethal dose) titer of the r268 virus, the nonvaccinated mice lost their body weights continuously and started to die from 6 days post-infection (dpi). In contrast, r268+133+158 saved all the mice from lethal challenge even with single immunization (Fig 2B). The single-immunized mice that received r268+133+158 lost approximately 20% and started to regain body weight from 8 dpi, and with double immunization, the mice lost only approximately 10% of their body weights (Fig 2C). However, single immunization with r268 protected only 20% to 30% of mice from lethal challenge (Fig 2B), and r268 exhibited protective efficacy only with double immunization (Fig 2C). Furthermore, the mouse antisera obtained by r268+133+158 double immunization produced the same or slightly higher HI titers against recombinant viruses harboring the HA and NA genes of 2013 to 2017 H7N9 strains of Yangtze River Delta and Pearl River Delta lineages (Table 2 and S4 Fig). These results indicate that r268+133+158 has the advantage of immunogenic breadth, whereas r268 induced reduced titers of HI-reactive antibodies to 2017 H7N9 isolates (Table 2).

**Table 2:** Immunogenic breadth of candidate vaccine viruses against 2013–2017 H7N9 strains.

Year	Virus^†^	Geometric mean HI-reactive antibody titers of double immunized mouse antiserum							
		α-r268		α-r268+133+158		α-rJS01		α-rJS01+133+158	
		Vaccine only	w/ alum adjuvant	Vaccine only	w/ alum adjuvant	5 MLD_50_ challenge	10 MLD_50_ challenge	5 MLD_50_ challenge	10 MLD_50_ challenge
2013	r268	40	80	80	160	80	160	320	320
	r268+133+158	28.28	40	40	80	40	40	320	160
	rJS01	n.d.^‡^	n.d.	n.d.	n.d.	40	40	80	160
	rJS01+133+158	n.d.	n.d.	n.d.	n.d.	20	20	40	40
2014	rAH887	56.57	80	80	113.14	80	160	320	320
2015	rGD002	80	160	80	160	80	160	320	320
	rGD351	56.57	40	40	113.14	320	320	640	640
2016	rGD060	80	160	80	160	160	160	320	320
	rGD061	80	160	80	160	160	160	320	320
	rGD923	80	160	80	160	80	160	320	320
2017	rZJ003	28.28	80	40	160	80	160	640	640
	rZJ005	28.28	80	80	113.14	80	160	320	320
	rHN287	28.28	80	80	80	80	80	160	160
	rFJ151	20	80	80	113.14	40	80	160	160

^†^Abbreviations of 6:2 recombinant viruses for Yangtze River Delta lineage strains are A/Anhui/01887/2014, AH887; A/Zhejiang/3/2017, ZJ003; A/Zhejiang/5/2017, ZJ005; A/Hunan/02287/2017, HN287; and A/Fujian/02151/2017, FJ151. The abbreviations for Pearl River Delta lineage strains are A/Guangdong/15SF002/2015, GD002; A/Environment/Guangdong/22351/2015, GD351; A/Guangdong/60060/2016, GD060; A/Guangdong/60061/2016, GD061; and A/Guangdong/60923/2016, GD923). Given the HA amino acid sequences of these viral strains, residue 240 is the only glycosite around the globular head region, except for the HA of rGD923 harboring additional NLG at residue 165 and the HA of rGD351 harboring additional NLG at residue 224.

^‡^n.d., not determined. Serum of naïve mice, used as a negative control, resulted in no HI reaction.

### NLG modification strategy for the development of efficacious avian influenza virus vaccines

**Fig 3:**
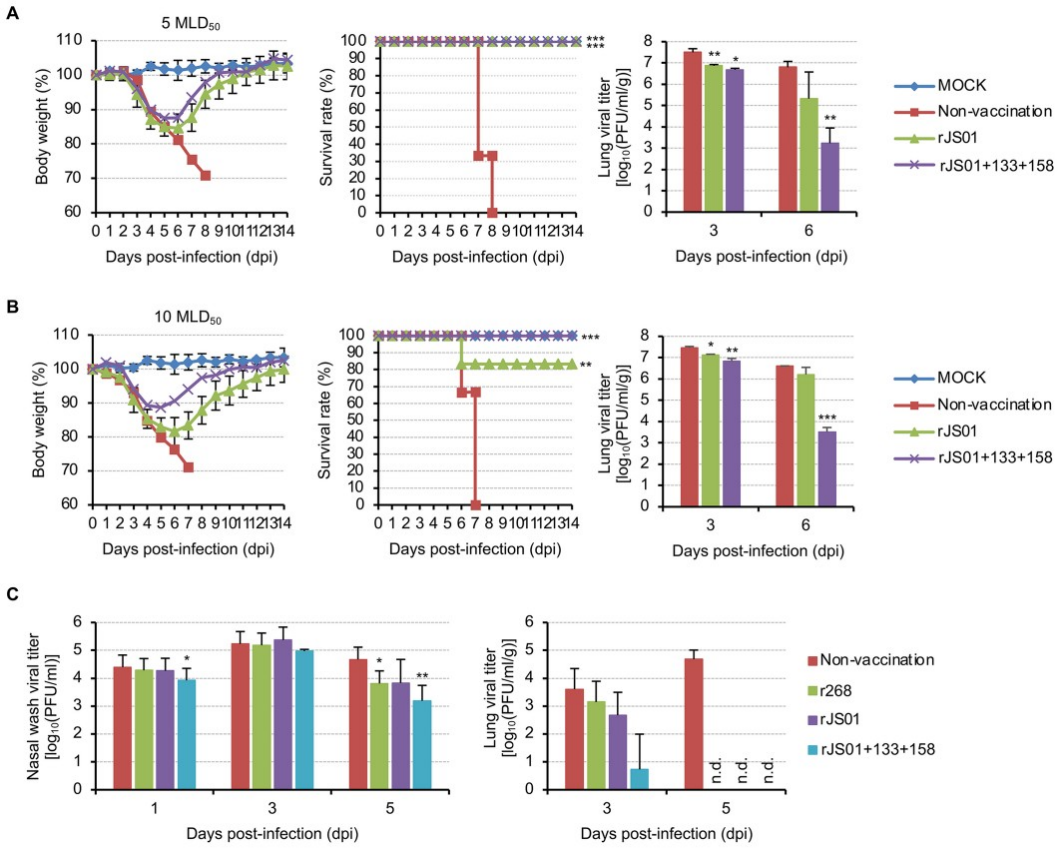
Feasibility assessment of the HA NLG modification strategy to design avian influenza vaccines in mice and ferrets. Body weight changes and survival rates of the PBS-injected (nonvaccinated) and vaccinated mice and viral titers in their lungs were analyzed for 14 days after lethal viral challenge (**A**, 5 MLD_50_ challenge and **B**, 10 MLD_50_ challenge). The mice in the mock group were vaccinated and challenged with PBS. Viral titers in the nasal washing samples and the lungs of the PBS-injected (nonvaccinated) and priming-boosting vaccinated ferrets were determined after viral challenge (**C**). The statistical significance of the differences in viral titers relative to those in the nonvaccinated mice or ferrets was assessed by Student *t* test (*, *P* < 0.05; **, *P* < 0.01; and ***, *P* < 0.001). Error bar denotes SD. Please see S3 Data for the numerical values used in Fig 3. HA, hemagglutinin; MLD_50_, 50% mouse lethal dose; n.d., not detected; NLG, N-linked glycosylation.

To confirm whether the addition of NLG to the HA globular head region increases the efficacy of avian influenza vaccines, we applied our strategy to another H7N9 strain, A/Shanghai/JS01/2013 (JS01; NCBI Taxonomy ID: 1395980) (S4 Fig) and developed rJS01 and rJS01+133+158 candidate vaccine viruses. In a mouse model, priming and boosting immunization with rJS01 or rJS01+133+158 reduced viral replication in the lungs against a 5 MLD_50_ titer viral challenge compared with the viral titers in nonvaccinated mice at 3 and 6 dpi, and both candidate vaccine viruses protected all the mice from lethal viral challenge (Fig 3A). Notably, rJS01+133+158, the candidate vaccine with 2 HA NLGs added, exhibited better efficacy than rJS01, as demonstrated with r268+133+158 (Fig 2B and 2C), and the improved efficacy of rJS01+133+158 was confirmed again even against a higher titer (10 MLD_50_) viral challenge. rJS01+133+158 still exhibited its efficacy in the lungs of challenged mice and saved all the mice from death, whereas rJS01 protected 5 out of the 6 mice (Fig 3B).

The applicability of our HA NLG modification strategy for the development of efficacious avian influenza vaccines was also successfully demonstrated in a ferret model. Compared with the vaccines having the WT HA (r268 and rJS01), rJS01+133+158 effectively suppressed viral replication in the upper and lower respiratory tracts of ferrets (Fig 3C). Furthermore, the ferret antiserum of rJS01+133+158 showed better reactivity against the heterologous viruses with more than 160 HI titers (against r267, 160; r268, 320; r268+133+158, 160; and rJS01, 160) than the antisera of the vaccines having the WT HA (r268 and rJS01) (S5 Fig). Similarly, the mouse antisera of rJS01+133+158 also showed much higher HI titers against the heterologous 2013 to 2017 H7N9 strains of the Yangtze River Delta and Pearl River Delta lineages than did rJS01 (Table 2). As demonstrated with r268+133+158, the addition of 2 HA NLGs appeared to compensate for the antigenic disparity between the vaccine with the WT HA (rJS01) and the heterologous 2013 to 2017 H7N9 strains.

### HA protein incorporation in the NLG modified vaccine viruses

**Fig 4:**
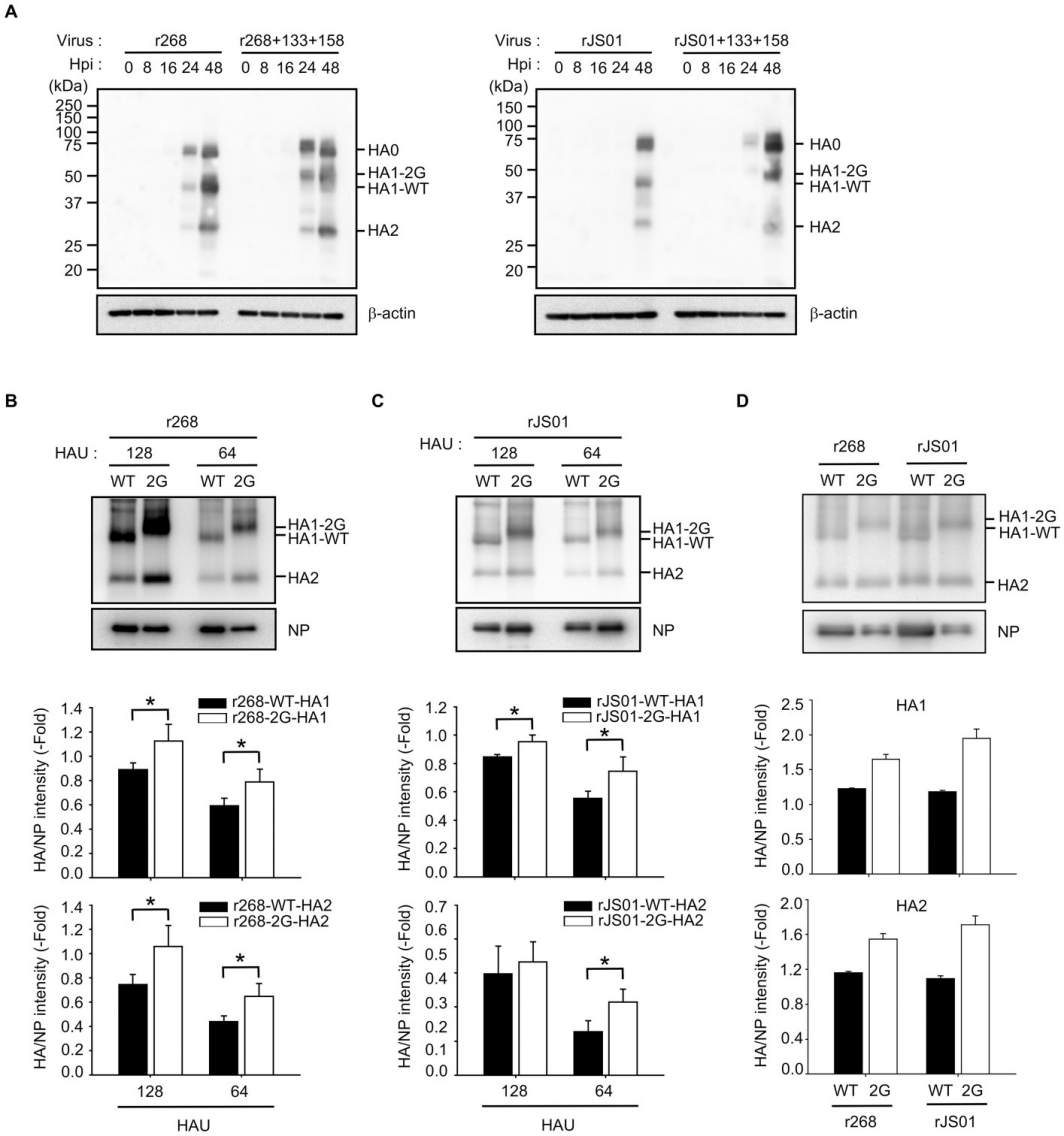
Comparison of HA contents incorporated in viral particles. Infected with r268, r268+133+158, rJS01, and rJS01+133+158, MDCK cells were collected for the comparison of HA protein expressions by western blot assays (**A**). The same MDCK cell supernatants collected at 48 hpi were analyzed for the comparison of HA protein contents incorporated into viral particles in 3 independent experiments (**B** and **C**). Given the lowest HA titers of each virus-infected cell supernatants, 128 HAU samples were used for western blotting. Given the intensity of WT HA protein expression (r268 and rJS01), HA expression of 2G viruses (r268+133+158 and rJS01+133+158) was quantified as fold differences. After quantified, 1 μg of the HA protein dose of the vaccine antigens was compared for their HA and NP protein contents in 2 independent experiments (**D**). 2G indicated 2 NLGs added at HA residues 133 and 158. The statistical significance of differences in HA protein expression was assessed by Student *t* test (*P < 0.05). n.s., no significance (*P* > 0.05). Error bar denotes SD. Please see S4 Data for the numerical values used in Fig 4. HA, hemagglutinin; HAU, hemagglutination unit; hpi, hours post-infection; MDCK, Madin-Darby canine kidney; NLG, N-linked glycosylation; NP, nucleoprotein; WT, wild-type.

Our results showed the better immunogenicity and in vivo efficacy of the NLG-mutant vaccine viruses (r268+133+158 and rJS01+133+158). With regard to the mode(s) of action of these NLG-mutant vaccine viruses, we next investigated the expression levels of HA proteins, which is one of the important factors determining the efficacy of influenza vaccines. When analyzed using MDCK cell lysates inoculated with r268, rJS01, and their respective NLG-mutants (r268+133+158 and rJS01+133+158), expression of HA1 and HA2 proteins of r268+133+158 and rJS01+133+158 increased much more than did that of WT, which appear to be consistent with the higher replication property of r268+133+158 in mammalian cells (Fig 1C–1E); at 24 hpi, the HA proteins of rJS01 were barely detected, whereas the HA proteins of rJS01+133+158 were detected in the cell lysates (Fig 4A). To determine how many HA proteins can be incorporated in each viral particle, we also analyzed HA expression using the same MDCK cell supernatants. For this analysis, we used 64 and 128 hemagglutination unit (HAU) of viral particles (S6 Fig) and compared the expression intensity of HA proteins given the expression levels of respective NP proteins in each HAU condition. Intriguingly, according to western blot results of 64 and 128 HAU of viral particles, HA1 and HA2 protein expression increased in r268+133+158 and rJS01+133+158 compared with in r268 and rJS01, respectively (Fig 4B and 4C). To confirm again, we quantified the HA protein contents of r268, r268+133+158, rJS01, and rJS01+133+158 in a single radial immunodiffusion (SRID) assay (S7 Fig and S5 Table), and 1 μg of the HA protein dose of each vaccine antigen was used for the comparison of HA and NP protein contents in the western blot assay. When the intensity of HA versus NP proteins was compared (Fig 4D and S8 Fig), it was revealed again that the NLG mutant vaccine antigens (r268+133+158 and rJS01+133+158) retained higher contents of HA proteins (HA1 or HA2 protein regions) than the respective WT vaccine antigens (r268 and rJS01), as presented in Fig 4B and 4C. This might indicate the higher content of HA proteins in the viral particles of the NLG-mutant vaccine viruses than of WT, which may explain the better protective efficacy of r268+133+158 and rJS01+133+158 in in vivo challenge studies (Figs 2 and 3). Collectively, these results suggest the feasibility of HA NLG modification as a solid strategy to design influenza vaccines with enhanced efficacy.

## Discussion

In our study, we examined whether modifying HA NLG patterns improves the efficacy of H7N9 vaccine strains. Due to the low immunogenicity of H7N9 candidate vaccines [14–16], there have been several efforts to improve vaccine efficacy. The adaptation of the H7N9 vaccine virus by serial passaging in MDCK cells [33] and by using an adenoviral vector [34] are examples of such efforts, despite the setbacks of these approaches, such as the appearance of multiple mutations in various viral protein-encoding regions during adaptation and the hurdle of obtaining clinical approval for DNA vaccine constructs. To overcome these issues, we tested an HA NLG modification strategy [27,28].

Unexpectedly, the addition of NLG modifications to the HA head region decreased the HI-reactive and PRNT antibody titers to the parental rH7 virus (S4 Table). Specifically, the antisera raised to the mutant viruses harboring an NLG modification at HA residue 133 (rH7+133 and rH7+133+158) showed decreases in the antibody titers, whereas the antiserum raised to rH7+158 produced antibody titers similar to those obtained with parental rH7 virus (S4 Table). This effect may be caused by the loss of efficient HA binding to SA receptors because of the NLG at HA residue 133, which is located in the vicinity of the receptor-binding site (RBS) (S1 Fig). Compared with the location of residue 133 in the HA structure, residue 158 is located at the upper tip of the HA head region, which might be one reason for the HI titer differences of the antisera induced by the viruses harboring the additional NLG either at residue 133 or at residue 158. With regard to the transmissibility of the H7N9 virus, however, NLG modifications on both residues appeared to result in deleterious effects [35]. Only the functionally well-matched NA might compensate for viral fitness and erase the deleterious effects on viral transmission [35,36].

Given the aim of our study, we focused on the rH7+133+158 virus because of its efficient replication in cells (Fig 1C–1E) and the immunogenic potential of broad-spectrum antibody responses across the other NLG-mutant viruses (Fig 1F). To further evaluate the immunogenicity of the rH7+133+158 virus in an inactivated vaccine formulation, we developed the H7N9 vaccine virus r268+133+158. Consistently, the r268+133+158 candidate vaccine virus elicited much higher HI titers than r268 in all 4 immunized groups of mice, regardless of adjuvant supplementation (Table 1). Additionally, immunization with r268+133+158 reduced viral replication in the lungs of mice and protected them from lethal viral challenge (Fig 2B and 2C). Moreover, a double immunization strategy with alum adjuvant removed traces of viral replication from the lungs at 3 days and 5 days after viral challenge (Fig 2C).

When compared with viral replication titers in the lungs and survival rates of mice immunized with r268 (Fig 2B and 2C), the in vivo immunogenicity and protective efficacy of r268+133+158 appeared remarkable. As mentioned above, NLG modifications at HA residues 133 and 158 reduced the immunogenicity of rH7 (S4 Table). However, when accompanied by the N9 NA protein of A/Anhui/1/2013 and the 6 internal genes of the PR8 vaccine donor, r268+133+158 exhibited enhanced effects, with or without adjuvant supplementation, compared with r268 (Fig 2B and 2C and Table 1). The masking effects of the HA NLG modification might also benefit the efficacy of r268+133+158. As suggested regarding the HA of human H1N1 strains before the 2009 pandemic [28], NLG modifications in a certain HA residue might reduce antigenic variation among different strains, whereas residues free from the NLG-mediated constraints might exhibit more immunogenic properties than those with NLG modifications. Hence, given that NLG modifications in the HA protein can arise in response to vaccine-guided immunity in hosts, the NLG-mediated antigenic masking effects might provide better potential as a vaccine antigen for circulating viral variants. This is well demonstrated in the antigenicity comparison test (using the HI assay) between r268+133+158 and recombinant H7N9 viruses harboring the HA and NA genes of 2013 to 2017 H7N9 strains (Table 2).

Although the enhanced efficacy of our candidate vaccine virus was well demonstrated in vitro and in vivo, we still need to confirm the general applicability of the HA NLG modification strategy for the development of efficacious avian influenza vaccines. To this end, we developed another H7N9 candidate vaccine virus (rJS01+133+158) using the same HA NLG modification strategy. As demonstrated with the in vivo efficacy of r268+133+158 (Fig 2B and 2C), rJS01+133+158 exhibited better protective efficacy against the 2 different doses of lethal viral challenge in mice than rJS01 harboring the WT HA (Fig 3A and 3B), and even in a ferret model, rJS01+133+158 greatly reduced viral replication in the lower and upper respiratory tracts (Fig 3C). Furthermore, in the antigenicity comparison test, the antiserum of rJS01+133+158-immunized mice showed even higher HI-reactive antibody titers against 2013 to 2017 H7N9 strains than that of r268+133+158-immunized mice (Table 2), which also indicates the importance of not only the HA residues of NLG modification but also the selection of an appropriate vaccine strain.

We selected HA residues 133 and 158 for NLG modification based on the evolutionary history of H7 HA NLGs (S1 Table), and it is interesting that HA residue 158 is included in the T cell epitope region (YAEMKWLLSNTDNAAFPQ; located in HA residues 148 to 163), which was suggested to be one of the human-like genetic sequences in the H7N9 genomic segments [37,38]. This might explain the possibility of NLG modification at HA residue 158 to change the quality of the T cell epitope and subsequently affect immune responses. Although the results of HI-reactive antibody titers were determined only using the antisera of animal models, our HA NLG modification approach might have merit for further application in vaccine development not only against emerging avian influenza viruses but also against human seasonal influenza viruses. This is because HA NLG modification may increase the content of HA proteins incorporated into viral particles of other (sub)type vaccine viruses (Fig 4), a property that can be applied for the refinement of vaccine viruses with poor immunogenicity and reduced efficacy, as demonstrated in our challenge studies using mice and ferrets (Figs 2 and 3). Moreover, from the viewpoint of the vaccine industry, this HA NLG modification strategy may have another merit to prompt high-yielding vaccines (Figs 2 and 4), which can replenish the short vaccine supply resulting from the suboptimal growth of human vaccine antigens in certain seasons. Considering all the evidence, we suggest that the addition of 2 NLG modifications on HA may be a feasible approach for fine-tuning the balance between viral infectivity and the immunogenicity of candidate vaccine viruses, and the HA NLG modification strategy may provide a solution to the unsatisfactory efficacy of avian influenza vaccines and help us to prepare against the pandemic potential of avian influenza viruses.

## Materials and methods

### Ethical approval

This study was conducted in strict accordance with the recommendations from the Guide for the Care and Use of Laboratory Animals of the Animal and Plant Quarantine Agency of Korea. The protocols for mouse, guinea pig, and fertilized chicken egg experiments were approved by the Institutional Animal Care and Use Committee (IACUC) of Korea University College of Medicine (permit number: KUIACUC-2014-225, 249). Because of biosecurity concerns regarding genetic modifications that may alter viral characteristics of the avian-origin H7N9 virus, all the experimental steps handling H7N9 vaccine viruses were reviewed by the IACUC of Korea University College of Medicine (permit number: KUIBC-15-7-A-1) and performed inside the BSL3 facility at our college that has been certified by the Korean government since 2014 (certification number: KCDC-14-3-05).

### Viruses and cells

Influenza A/Korea/01/2009 (pandemic (H1N1) 2009) virus was provided by Korea Disease Control and Prevention Agency (Cheongju, Chungcheongbuk-do, Republic of Korea). The influenza H7N9 vaccine viruses, A/Anhui/1/2013 (6:2 virus, r268) and A/Shanghai/2/2013 (6:2 virus, r267), were provided to Il Yang Pharmaceutical (Yongin, Republic of Korea) by National Institute for Biological Standards and Control (Hertfordshire, United Kingdom). After purification using a plaque assay in MDCK cells, each virus was propagated in fertile chicken eggs for 48 to 72 hours. MDCK, human embryonic kidney (293T), human lung epithelial (A549), and African green monkey kidney (Vero) cells were obtained from the American Type Culture Collection (Manassas, Virginia, United States of America) and maintained in the appropriate medium (MDCK cells: Minimum Essential Medium Eagle with Earle’s BBS (MEME), Lonza, Basel, Switzerland; 293T, A549, and Vero cells: Dulbecco’s Modified Eagle Medium (DMEM), Gibco, Waltham, Massachusetts, USA) supplemented with 10% fetal bovine serum and antibiotics at 37 °C and 5% CO_2_.

### Plaque assay

To determine replication titers or to compare plaque phenotypes of viruses, a plaque assay was performed in MDCK cells. Briefly, each virus was diluted 10-fold in medium, and MDCK cells prepared in a 6-well plate were infected with the diluted virus at 37 °C for 1 hour. After the inoculum was discarded, the cells were washed 3 times with phosphate-buffered saline (PBS) and overlaid with medium containing 0.2% agar and 1 μg/ml L-(tosylamido-2-phenyl) ethyl chloromethyl ketone (TPCK)-treated trypsin. After approximately 72 hours of incubation at 37 °C, the cells were stained with crystal violet, and viral titers were determined by counting the number of plaques.

### HA NLG pattern analysis of H7-subtype viruses

A total of 1,361 HA-encoding nucleotide sequences from H7-subtype viruses (H7N1, *n* = 132; H7N2, *n* = 200; H7N3, *n* = 426; H7N7, *n* = 253; and H7N9, *n* = 350) were downloaded from the NCBI database using the Influenza Virus Resource website (http://www.ncbi.nlm.nih.gov/genomes/FLU/FLU.html) and used for NLG pattern analysis (Table 1). The HA-encoding sequences were first aligned and then converted into the amino acid sequence set using AliView (v1.17.1) and screened for the presence of NLG sequons of “N-Xaa-S/T” (where Xaa would be any amino acids except proline) using the N-GlycoSite website (http://www.hiv.lanl.gov/content/sequence/GLYCOSITE/glycosite.html).

### Plasmid-based reverse genetics

The genes encoding the HA and NA of influenza A/Anhui/1/2013 and A/Shanghai/JS01/2013 were commercially synthesized (Cosmogenetech, Seoul, Republic of Korea) and used for NLG modification. For the addition or removal of an NLG modification on HA, we mutated the nucleotide residues encoding the respective glycosylation sites in HA and verified the substitutions via commercial genetic sequence analysis (Cosmogenetech). The 4 plasmids that encoded each of the NLG-mutant HAs from the influenza A/Anhui/1/2013 virus were constructed using ambisense pDZ plasmids and used to generate 7:1 recombinant viruses on the genetic backbone (PB2, PB1, PA, NP, NA, M, and NS genes) of the influenza A/Korea/01/2009 strain, as described previously [39]. Briefly, the genetic plasmids, including the HAs with certain NLG modifications, were transfected into cocultured 293T and MDCK cells. After 48 to 72 hours, the cell supernatants were tested in a hemagglutination assay using 0.5% (vol/vol) turkey red blood cells (tRBCs), and the positive sample was inoculated into MDCK cells for plaque purification. Purified viral clones were then inoculated into 10-day-old fertilized chicken eggs for propagation. The genetic sequences of the rescued and propagated viruses were confirmed by reverse transcription polymerase chain reaction (RT-PCR). For subsequent analyses, we used the egg-grown viruses with confirmed sequences.

### Hemagglutination (HA) assay

To determine the HA titer of each virus, 50 μl of virus was 2-fold diluted in PBS and then allowed to agglutinate with the same volume of 0.5% (vol/vol) tRBCs at 4 °C for 45 minutes. The reciprocal of the highest dilution of HA-RBC agglutination was determined as the HA titer of each virus.

### Western blot assay

To confirm the biochemical usage of the NLG modification in the HA of each mutant virus, MDCK cells were inoculated at a multiplicity of infection (MOI) of 3. Sixteen hours later, the cells were treated with radioimmunoprecipitation assay buffer (Sigma, St. Louis, Missouri, USA), and the proteins in the lysates were separated by sodium dodecyl sulfate polyacrylamide gel electrophoresis (SDS-PAGE) and transferred to a polyvinylidene fluoride membrane. Polyclonal antibodies from guinea pigs infected with the rH7 virus were used as the primary antibody to detect the HA protein. The primary antibody (for HA, H7-Ab-1402; Center for Biologics Evaluation and Research, CBER, Food and Drug Administration, Silver Spring, Maryland, USA, and for NP, mouse polyclonal antibodies generated with the double immunization of r268 vaccine virus) was visualized after incubation with a horseradish peroxidase (HRP)-conjugated secondary antibody specific for guinea pig IgG (KPL, Gaithersburg, Maryland, USA) and a chemiluminescent substrate (Thermo Fisher Scientific, Waltham, Massachusetts, USA). On the same blots, β-actin was then detected as a loading control, using a mouse monoclonal antibody (Sigma).

To compare HA protein expressions, MDCK cells were infected with r268, r268+133+158, rJS01, and rJS01+133+158 at an MOI of 0.001. The cells were then collected at 8, 16, 24, and 48 hpi and processed for western blotting, as described above. To compare the HA protein contents incorporated into viral particles, HA titers of the same MDCK cell supernatants collected at 48 hpi were determined by the HA assay. Given the lowest HA titers of each virus-infected cell supernatants, 128 HAU samples were prepared and used for HA protein content analysis by western blotting. HA and NP proteins were detected by the primary and the HRP-conjugated secondary antibodies. Immune-reactive bands were visualized with an enhanced chemiluminescence reagent kit (AbFrontier, Seoul, Republic of Korea) and quantified by a ChemiDoc imaging system (Bio-Rad Laboratories, Hercules, California, USA).

### Growth kinetics of the NLG-mutant viruses in mammalian cells

For replication analysis, MDCK, A549, and Vero cells were inoculated with the virus at an MOI of 0.001. After 1 hour, the cells were washed with the appropriate medium (for MDCK cells: MEME, Lonza; for A549 and Vero cells: DMEM, Thermo Fisher Scientific) containing 0.3% bovine serum albumin (BSA) and TPCK-treated trypsin (1 μg/ml). The supernatants were then collected at each indicated time point and used for viral titration by the plaque assay in MDCK cells.

### Hemagglutination inhibition (HI) assay

Double-immunized animal antisera were used against H7N9 avian influenza virus strains. Briefly, guinea pig antisera collected after priming and boosting immunization were treated with a receptor-destroying enzyme (RDE, NA from *Vibrio cholerae*; Denka Seiken, Japan) before use. Four HA units of virus were incubated with the 2-fold–diluted antiserum in 96-well plates at 37 °C for 1 hour. After incubation, 50 μl of 0.5% (vol/vol) tRBCs was added to each well, and the HI titers were recorded after incubation for another 45 minutes at room temperature. Serum from naïve guinea pigs was used as a control. Primed only and primed-boosted mice and primed-boosted ferret antisera used for the HI or PRNT assay were collected immediately before virus challenge and were treated with RDE, as indicated above.

### Plaque-reduction neutralization test (PRNT)

To determine the titers of neutralizing antibodies, 100 PFU of a virus was incubated with 2-fold–diluted primed-boosted guinea pig antisera at 37 °C. After 1 hour of incubation, MDCK cells were inoculated with each virus–antiserum mixture for the plaque assay. The PRNT titer was then determined based on the titer that showed a 50% reduction in the number of viral plaques compared to that of a PBS-treated control. Serum from naïve guinea pigs was also used as a control.

### Candidate vaccine virus preparation

As described above, the genetic plasmids encoding the NLG-modified HA and the original NA of influenza A/Anhui/1/2013 (H7N9 human isolate and WHO-recommended vaccine strain) and A/Shanghai/JS01/2013 were constructed into the same pDZ plasmid. Six internal gene plasmids (PB2, PB1, PA, NP, M, and NS) of the human vaccine donor, influenza A/Puerto Rico/8/34 (PR8, H1N1 virus), were also constructed after RT-PCR of the r268 virus. The genetic sequences of these plasmids were confirmed by commercial sequencing before use. H7N9 candidate vaccine viruses (r268+133, r268+158, r268-240, and r268+133+158; rJS01 and rJS01+133+158) were then generated by using the HA and NA plasmids of A/Anhui/1/2013 and A/Shanghai/JS01/2013, respectively, upon the PR8 vaccine donor.

Of candidate vaccine viruses, r268, r268+133+158, rJS01, and rJS01+133+158 were inactivated for vaccine formulation. Briefly, after propagation of the vaccine viruses in fertilized chicken eggs, the allantoic fluids were collected by differential centrifugation using a sucrose density gradient. The vaccine viruses were then split using Triton X-100 (AMRESCO, Cleveland, Ohio, USA) and inactivated by treatment with formaldehyde. The HA purity and contents of the vaccine antigens were then assessed by using SDS-PAGE and SRID assays, respectively. SDS-PAGE and SRID analyses of the vaccine bulk were performed as described in the generic protocol of WHO [40]. Briefly, the vaccine antigens were mixed with sample buffer and then heated to 95 to 100 °C for 3 to 5 minutes. Approximately 1 μg of protein samples was loaded on 12% Bis-acrylamide gels and run at 120 V in running buffer (Life Technologies, Waltham, Massachusetts, USA). The gel was stained with Oriole Fluorescent Gel Stain (Bio-Rad Laboratories). The total protein was determined by a Lowry assay. The r267 vaccine virus was also treated with the same procedures for a ferret study.

For the SRID assay, reference antigen (A/Shanghai/02/2013, H7N9, PR8-IDCDC-RG32A; provided by Center for Biologics Evaluation and Research, CBER, Food and Drug Administration) and reference antibody (to the HA of A/Shanghai/02/2013, H7N9, H7-Ab-1402; provided by CBER) were used (S9 and S10 Figs). Briefly, 1% agarose gel containing 10 μl/ml reference antibody was prepared, and 16 to 24 wells for vaccine antigens were made using hole puncher (4 mm in diameter). Each vaccine antigen that was treated with a detergent was diluted (25%, 50%, 75%, and 100%) using PBS (pH 7.2). The vaccine antigens were loaded in each well on the agarose gel, and the gel was maintained in 22 ± 2 °C for 18 to 24 hours. The agarose gel was then washed with PBS (pH 7.2) at least for 30 minutes and washed again with distilled water. After being dried, the agarose gel was stained with Coomassie brilliant blue to detect the antigen-antibody precipitation on the gel. Based on the diameters of the antigen-antibody precipitation ring, HA protein concentrations of vaccine antigens were estimated. The SRID reagents were calibrated before use according to the protocol endorsed by the WHO Expert Committee on Biological Standardization [41].

### NLG-modified vaccine strain yields in fertilized chicken eggs

For the analysis of the growth rates and HA titers of the candidate vaccine viruses expressing NLG-modified HA (r268+133, r268+158, r268-240, and r268+133+158), a 10^3^ PFU titer of each vaccine virus was inoculated into the allantoic cavity of 10-day-old fertilized chicken eggs. After 48 hours of incubation at 37 °C, the allantoic fluids were collected and used for virus titration by the plaque assay in MDCK cells or by the HA assay using 0.5% (vol/vol) tRBCs.

### Animal experiments

Guinea pig antisera were used in the HI and PRNT assays. Briefly, 2 guinea pigs per virus group were primed and boosted after a 2-week interval by intranasal infection with 10^5^ PFUs in 100 μl of each virus, after anesthetization with a combination of xylazine (Narcoxyl, InterVet, the Netherlands) and zoletil (Zoletil 50, Virvac, France). Two weeks later, blood was collected from the guinea pigs, and the antisera were treated with RDE (Denka Seiken) before use.

Before being immunized or infected, mice were anesthetized with a combination of xylazine and zoletil, as described for the guinea pig experiment above. Female 5-week-old BALB/c mice were immunized either once (primed) or twice (primed and boosted after a 2-week interval) intramuscularly with the vaccine strain (3 μg), with or without 0.1% alum adjuvant. Two weeks after the last immunization, the mouse antisera were collected and treated with RDE for use in the HI and PRNT assays. Serum from naïve mice was used as a control. For the assessment of viral replication in the mouse lungs, immunized mice per group were infected intranasally with 10^6^ PFU in 30 μl of the r268 virus, and their lungs were collected 3 days and 5 days after infection. The lungs of the infected mice were collected and then homogenized in 1 ml of 0.3% BSA-added MEME and centrifuged at 13,000 rpm for 10 minutes at 4 °C. The supernatant was then used for virus titration. For a formal challenge study, 10 mice per group were immunized (single or double) with r268 or r268+133+158 with or without 0.1% alum adjuvant. Two weeks after the last immunization, the mice were challenged with 5 MLD_50_ titer of the r268 virus and monitored for 14 days for their body weight changes. Mice that lost more than 25% of their body weight were considered experimental deaths and euthanized humanely. The vaccination-challenge study of rJS01 and rJS01+133+158 used 6 BALB/c mice per group and was performed with the same protocol above but without alum supplementation. Viral titers were determined using the lung specimens of the rJS01 and rJS01+133+158 vaccinated and challenged mice that were collected at 3 and 6 dpi (3 mice per group per day).

Ferrets (male and female, 18 to 22 weeks old; Wuxi Sangosho Biotechnology, Jiangsu, China) were used for vaccine efficacy assessment. Ferrets per group were primed and boosted with each candidate vaccine virus with a 2-week interval and were infected intranasally with a 10^6^ PFU titer of r268. Nasal washing samples were collected at 1, 3, and 5 dpi, and 3 ferrets per group were sacrificed at 3 and 5 dpi for virus titration by the plaque assay.

### Antigenicity comparison assessment

To evaluate the viral antigenicity between the candidate vaccine viruses and recent H7N9 strains, we commercially synthesized the HA and NA genes of 2014 to 2017 avian influenza H7N9 isolates (Yangtze River Delta lineage strains: A/Anhui/01887/2014, AH887; A/Zhejiang/3/2017, ZJ003; A/Zhejiang/5/2017, ZJ005; A/Hunan/02287/2017, HN287; and A/Fujian/02151/2017, FJ151 and Pearl River Delta lineage strains: A/Guangdong/15SF002/2015, GD002; A/Environment/Guangdong/22351/2015, GD351; A/Guangdong/60060/2016, GD060; A/Guangdong/60061/2016, GD061; and A/Guangdong/60923/2016, GD923) (Bionics, Seoul, Republic of Korea) that had been registered into the EpiFlu database of Global Initiative on Sharing All Influenza Data (http://platform.gisaid.org/epi3/frontend#275180), based on the evolutionary relationships of H7N9 HA genes that were reported by WHO (Analysis of recent scientific information on avian influenza A (H7N9) virus, as of February 10, 2017; http://www.who.int/influenza/human_animal_interface/avian_influenza/Annex1_Phylogenetic_tree_for_haemagglutinin_gene.pdf?ua=1&ua=1), and used for the generation of 6:2 recombinant viruses on the PR8 genetic backbone, as explained above, by reverse genetics. The recombinant viruses were propagated in embryonated chicken eggs and confirmed by sequence analysis before use. The mouse antisera obtained by double immunization that were diluted to make 40 homologous HI titers were also used for viral antigenicity comparison in the HI assay.

### Statistical analysis

The statistical significance of differences in the properties of viral replication in the cells and eggs was evaluated by a 1-way analysis of variance (ANOVA) test and confirmed by Tukey multiple comparison test. Survival graphs were prepared by Kaplan–Meier method and statistically analyzed with the Mantel–Cox log-rank test followed by the Gehan–Breslow–Wilcoxon test. To assess the statistically significant differences in the viral titers in the lungs of mice and in the nasal wash samples of ferrets, Student *t* test was used.

## Supporting information

S1 Fig Structural representation of potential glycosites located around the HA head region. Three potential NLG residues (133, 158, and 240) in HA1 region were indicated around the HA head region using the HA structure of A/Shanghai/01/2013 (PDB ID: 4LCX). Only 1 HA monomer was indicated with colors (light blue, HA1 and light pink, HA2). The NLG residues were colored with red, and the RBS was colored with dark blue. HA, hemagglutinin; NLG, N-linked glycosylation; RBS, receptor-binding site.
(TIF)

S2 Fig Raw data of western blot results in Fig 1A. HA protein expression of rH7 and its NLG mutant viruses was compared by the western blot assay. To support the results presented in Fig 1A, raw data files of for HA and β-actin proteins were presented.
(TIF)

S3 Fig Body weight changes and survival rates of mice infected with rH7 and its NLG mutant viruses. BALC/c mice (female, 5–6 weeks old) were infected at 10^5^ (**A**) and 10^6^ (**B**) PFU titers of rH7 and its NLG mutant viruses (rH7+133, rH7+158, rH7-240, rH7+133+158), and their body weight changes were monitored for 14 days. Mice losing more than 25% from original body weights (measured at 0 dpi) were considered experimentally dead, and based on these changes, survival rates were determined. Error bar denotes SD. Please see S5 Data for the numerical values used in S3 Fig. NLG, N-linked glycosylation; PFU, plaque-forming unit; SD, standard deviation.
(TIF)

S4 Fig Phylogenetic tree of H7N9 HAs. Using a Bayesian inference method implemented in BEAST (v1.10.4; GTR+I+Γ substitution and lognormal relaxed molecular clock models, and a Bayesian skygrid tree prior), the evolutionary relationship of H7N9 HAs (*n* = 350, downloaded from the GISAID EpiFlu database) was reconstructed. Colors of node circles represent posterior probability values, as indicated in the legend. The viral strains used in the antigenicity comparison tests in Table 2 are indicated with tangerine and aqua blue colors according to their respective genetic lineages (Yangtze River Delta and Pearl River Delta). The 2 candidate vaccine viruses (A/Anhui/1/2013 and A/Shanghai/JS01/2013) are colored with red. Given the HA amino acid sequences of these viral strains, residue 240 is the only glycosite around the globular head region, except for the HA of rGD923 harboring additional NLG at residue 165 and the HA of rGD351 harboring additional NLG at residue 224. Please see S6 Data for HA sequences used for the phylogenetic analysis in S4 Fig.
(PDF)

S5 Fig Immune responses of ferret antisera against the NLG-mutant vaccine viruses. The antisera (a-r268, a-rJS01, and a-rJS01+133+158) of ferrets were used for the HI assay to examine their immunogenic profiles against H7N9 candidate vaccines (r267, r268, r268+133+158, rJS01, and rJS01+133+158). The ferrets in the nonvaccinated mice were immunized with PBS. Error bar denotes SD. Please see S7 Data for the numerical values used in S5 Fig. HI, hemagglutination inhibition; NLG, N-linked glycosylation; PBS, phosphate-buffered saline; SD, standard deviation.
(TIF)

S6 Fig Data of HA assay and western blot results in Fig 4A–4C. To support the results presented in Fig 4A–4C, raw data files of the HA (**A**) and western blot (**B**) assays were presented. Indications for lane and experimental conditions were also presented. Raw data of Fig 4A were obtained from the MDCK cell lysates samples and those of Fig 4B and 4C from the MDCK cell supernatants. 2G indicated 2 NLGs added at HA residues 133 and 158. HA, hemagglutinin; MDCK, Madin-Darby canine kidney; NLG, N-linked glycosylation.
(TIF)

S7 Fig Measurement of HA protein contents in the vaccine antigens by the SRID assay. To quantify the HA protein contents in each vaccine antigen, an antigen-antibody precipitation reaction was performed in the SRID assay. PR8-IDCDC-RG32A of A/Shanghai/02/2013 was used as a positive control antigen, and all antigens were reacted with a reference antibody, CNER H7-Ab-1302 (anti-HA antibody of A/Shanghai/02/2013). The quantified HA protein contents were presented in S5 Table.
(PDF)

S8 Fig Raw data of western blot results in Fig 4D. To support the results presented in Fig 4D, raw data files of the western blot assay were presented. Raw data were obtained from the quantified (1 mg concentration of the HA protein dose) vaccine antigens. 2G indicated 2 NLGs added at HA residues 133 and 158.
(TIF)

S9 Fig Product circular of a reference antigen used in the SRID assay. Specifications of a reference antigen were provided in the product circular.
(PDF)

S10 Fig Product circular of a reference antibody used in the SRID assay. Specifications of a reference antibody were provided in the product circular.
(PDF)

S1 Table Frequency of NLG modifications on HAs from selected H7-subtype viruses. (DOCX)

S2 Table HA NLG signatures of the mutant viruses and their viral titers. (DOCX)

S3 Table Pathogenicity of the HA NLG mutant viruses in BALB/c mice. (DOCX)

S4 Table Pairwise HI (PRNT) titers of guinea pig antisera. (DOCX)

S5 Table HA protein concentration included in the vaccine antigens. (DOCX)

S1 Data Numerical values in Fig 1. Sheet 1: Viral growth kinetics data in MDCK cells. Sheet 2: Viral growth kinetics data in A549 cells. Sheet 3: Viral growth kinetics data in Vero cells. Sheet 4: Pairwise HI and PRNT titers of vaccinated guinea pig sera against rH7 NLG mutant viruses.
(XLSX)

S2 Data Numerical values in Fig 2. Sheet 1: The growth rates and HA contents of the candidate vaccine viruses in embryonated chicken eggs. Sheet 2: Body weight changes and survival rates of the vaccinated mice (r268 and r268+133+158) after lethal challenge. Sheet 3: Lung viral titers of the vaccinated mice (r268 and r268+133+158) after lethal challenge.
(XLSX)

S3 Data Numerical values in Fig 3. Sheet 1: Body weight changes and survival rates of the vaccinated mice (rJS01 and rJS01+133+158) after lethal challenge after lethal challenge. Sheet 2: Lung viral titers of the vaccinated mice (rJS01 and rJS01+133+158) after lethal challenge. Sheet 3: Nasal wash viral titers of the vaccinated ferrets (rJS01 and rJS01+133+158) after challenge. Sheet 4: Lung viral titers of the vaccinated ferrets (rJS01 and rJS01+133+158) after challenge.
(XLSX)

S4 Data Numerical values in Fig 4. Sheet 1: Quantitative data of the comparison of HA protein expression based on the HAU (64 and 128) values. Sheet 2: Quantitative data of the comparison of HA protein expression based on the SRID-measured HA contents.
(XLSX)

S5 Data Numerical values in S3 Fig. Sheet 1: Body weight changes and survival rates of the mice infected with rH7 NLG mutant viruses.
(XLSX)

S6 Data HA sequences of H7N9 viruses used for the phylogenetic analysis in S4 Fig. The HA sequences used in this study were provided in a fasta format.
(TXT)

S7 Data Numerical values in S5 Fig. Sheet 1: HI titers of the vaccinated ferret antisera against r268, rJS01, and their respective NLG mutant viruses.
(XLSX)

## Abbreviations

ANOVA: analysis of variance
Asn: asparagine
BSA: bovine serum albumin
CBER: Center for Biologics Evaluation and Research
DMEM: Dulbecco’s Modified Eagle Medium
dpi: days post-infection
GMT: geometric mean titer
HA: hemagglutinin
HAU: hemagglutination unit
HI: hemagglutination inhibition
HPAI: highly pathogenic avian influenza
hpi: hours post-infection
HRP: horseradish peroxidase
IACUC: Institutional Animal Care and Use Committee
IAV: influenza A virus
MDCK: Madin-Darby canine kidney
MEME: Minimum Essential Medium Eagle
MLD50: 50% mouse lethal dose
MOI: multiplicity of infection
NA: neuraminidase
NCBI: National Center for Biotechnology Information
NLG: N-linked glycosylation
NP: nucleoprotein
PBS: phosphate-buffered saline
PFU: plaque-forming unit
PRNT: plaque-reduction neutralization test
RBS: receptor-binding site
RDE: receptor-destroying enzyme
RT-PCR: reverse transcription polymerase chain reaction
SA: sialic acid
SD: standard deviation
SDS-PAGE: sodium dodecyl sulfate polyacrylamide gel electrophoresis
SRID: single radial immunodiffusion
TPCK: L-(tosylamido-2-phenyl) ethyl chloromethyl ketone
tRBC: turkey red blood cell
WT: wild-type
