# Supplementary figures and images for: Glycosylation generates an efficacious and immunogenic vaccine against H7N9 influenza virus
Source: PLoS Biol. 2020 Dec 23;18(12):e3001024. doi: 10.1371/journal.pbio.3001024 (PMC7757820; doi:10.1371/journal.pbio.3001024)

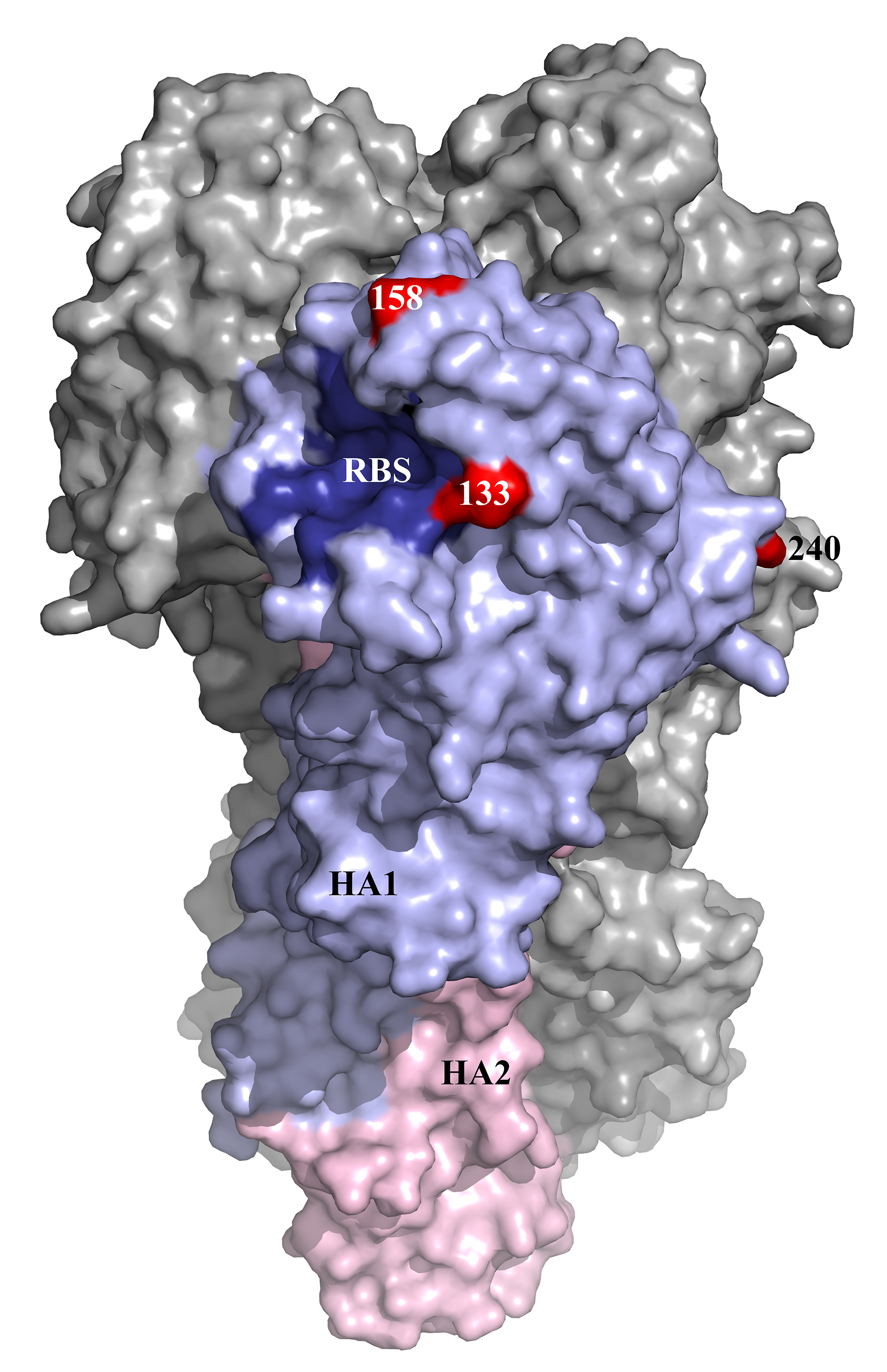

Supplement: S1 Fig — Three potential NLG residues (133, 158, and 240) in HA1 region were indicated around the HA head region using the HA structure of A/Shanghai/01/2013 (PDB ID: 4LCX). Only 1 HA monomer was indicated with colors (light blue, HA1 and light pink, HA2). The NLG residues were colored with red, and the RBS was colored with dark blue. HA, hemagglutinin; NLG, N-linked glycosylation; RBS, receptor-binding site. (TIF) [file pbio.3001024.s001.tif]

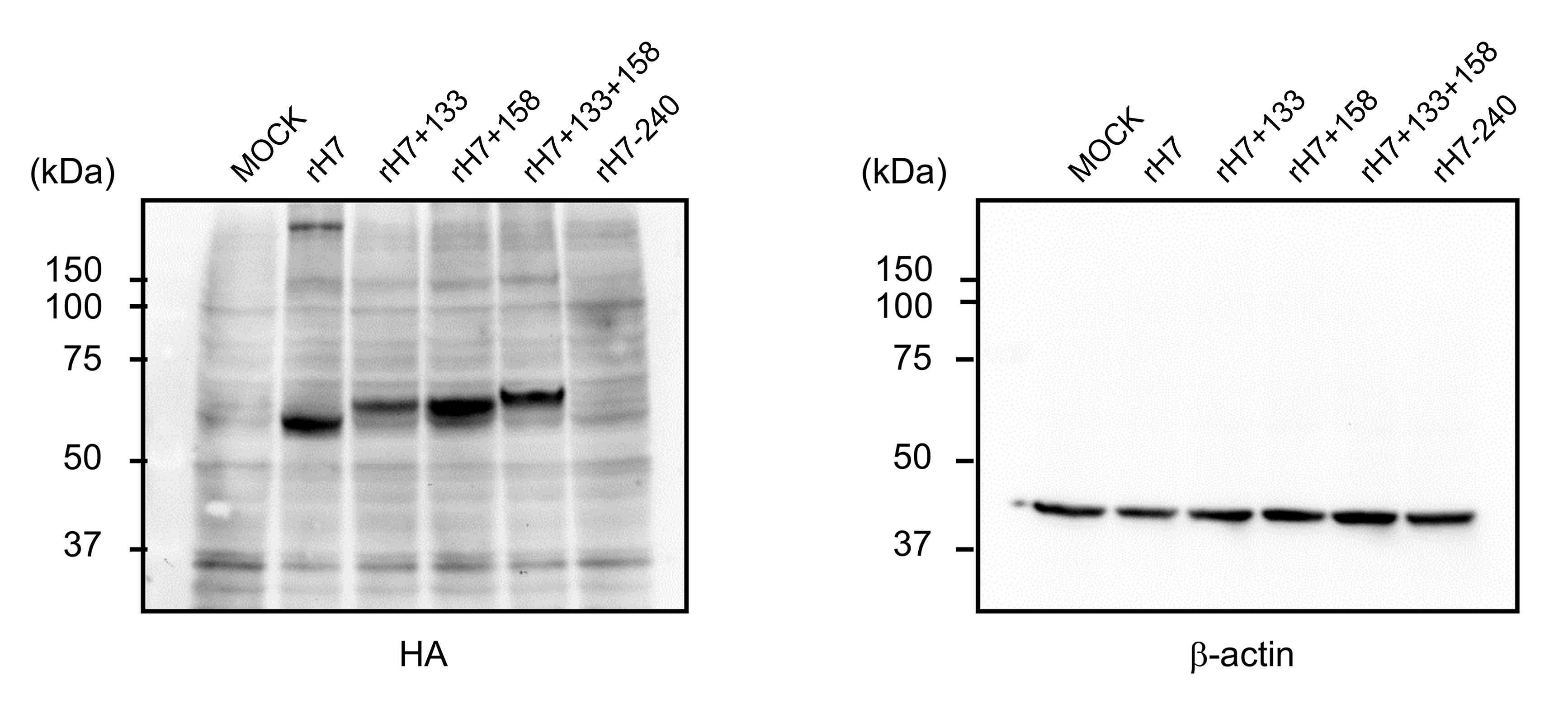

Supplement: S2 Fig — HA protein expression of rH7 and its NLG mutant viruses was compared by the western blot assay. To support the results presented in Fig 1A, raw data files of for HA and β-actin proteins were presented. (TIF) [file pbio.3001024.s002.tif]

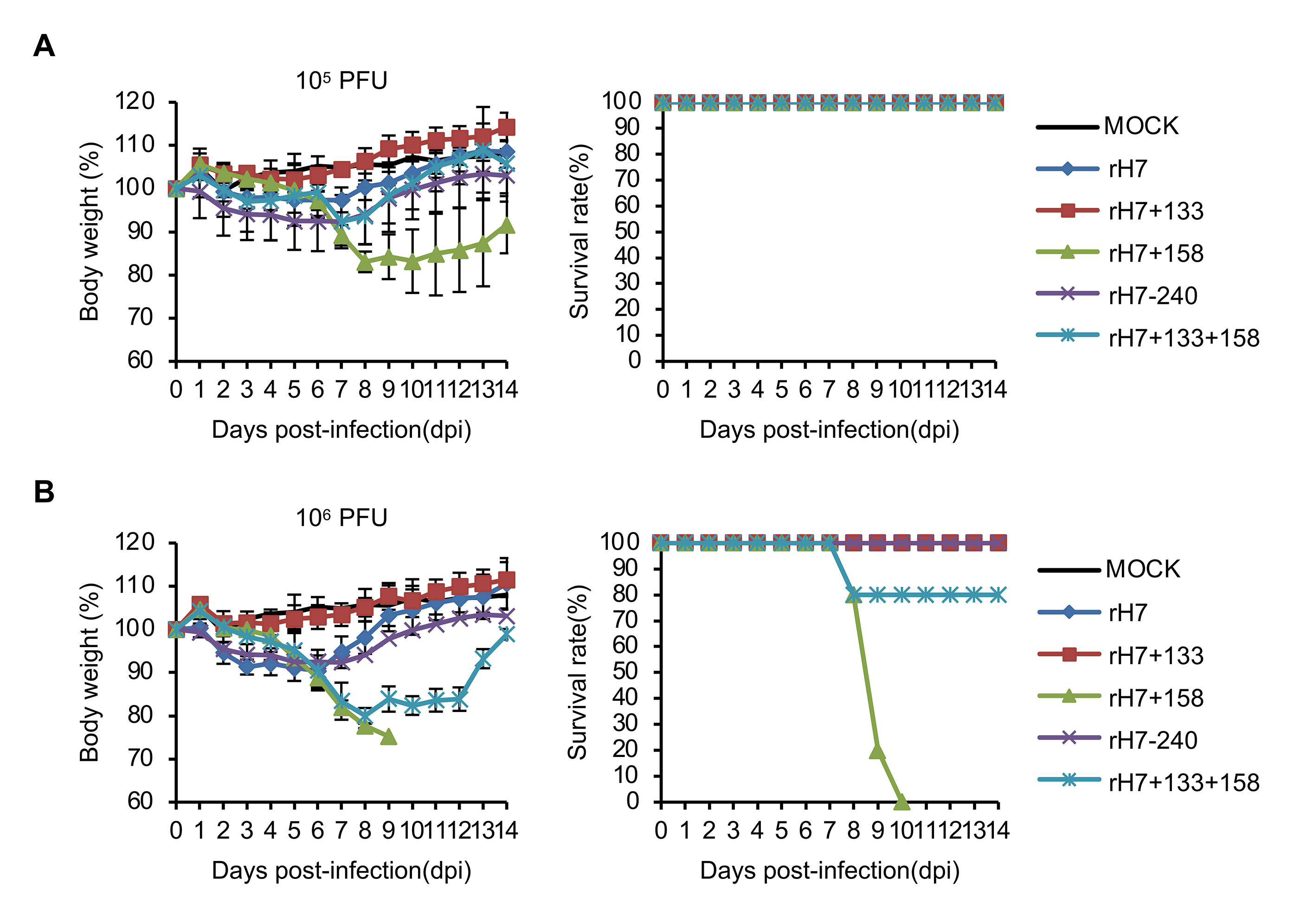

Supplement: S3 Fig — BALC/c mice (female, 5–6 weeks old) were infected at 105 (A) and 106 (B) PFU titers of rH7 and its NLG mutant viruses (rH7+133, rH7+158, rH7-240, rH7+133+158), and their body weight changes were monitored for 14 days. Mice losing more than 25% from original body weights (measured at 0 dpi) were considered experimentally dead, and based on these changes, survival rates were determined. Error bar denotes SD. Please see S5 Data for the numerical values used in S3 Fig. NLG, N-linked glycosylation; PFU, plaque-forming unit; SD, standard deviation. (TIF) [file pbio.3001024.s003.tif]

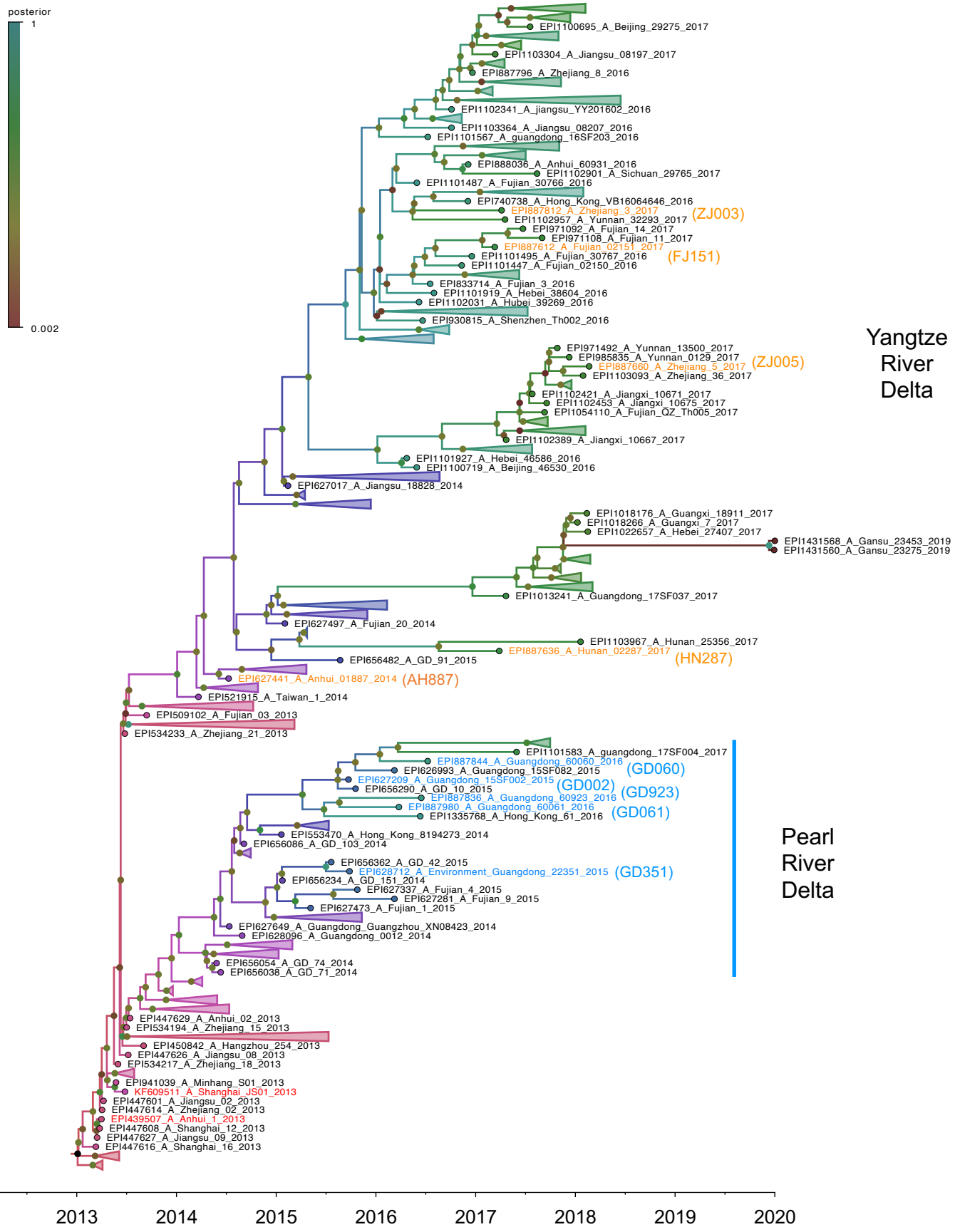

Yangtze  
River  
Delta

Pearl  
River  
Delta

Supplement: S4 Fig — Using a Bayesian inference method implemented in BEAST (v1.10.4; GTR+I+Γ substitution and lognormal relaxed molecular clock models, and a Bayesian skygrid tree prior), the evolutionary relationship of H7N9 HAs (n = 350, downloaded from the GISAID EpiFlu database) was reconstructed. Colors of node circles represent posterior probability values, as indicated in the legend. The viral strains used in the antigenicity comparison tests in Table 2 are indicated with tangerine and aqua blue colors according to their respective genetic lineages (Yangtze River Delta and Pearl River Delta). The 2 candidate vaccine viruses (A/Anhui/1/2013 and A/Shanghai/JS01/2013) are colored with red. Given the HA amino acid sequences of these viral strains, residue 240 is the only glycosite around the globular head region, except for the HA of rGD923 harboring additional NLG at residue 165 and the HA of rGD351 harboring additional NLG at residue 224. Please see S6 Data for HA sequences used for the phylogenetic analysis in S4 Fig. (PDF) [file pbio.3001024.s004.pdf]

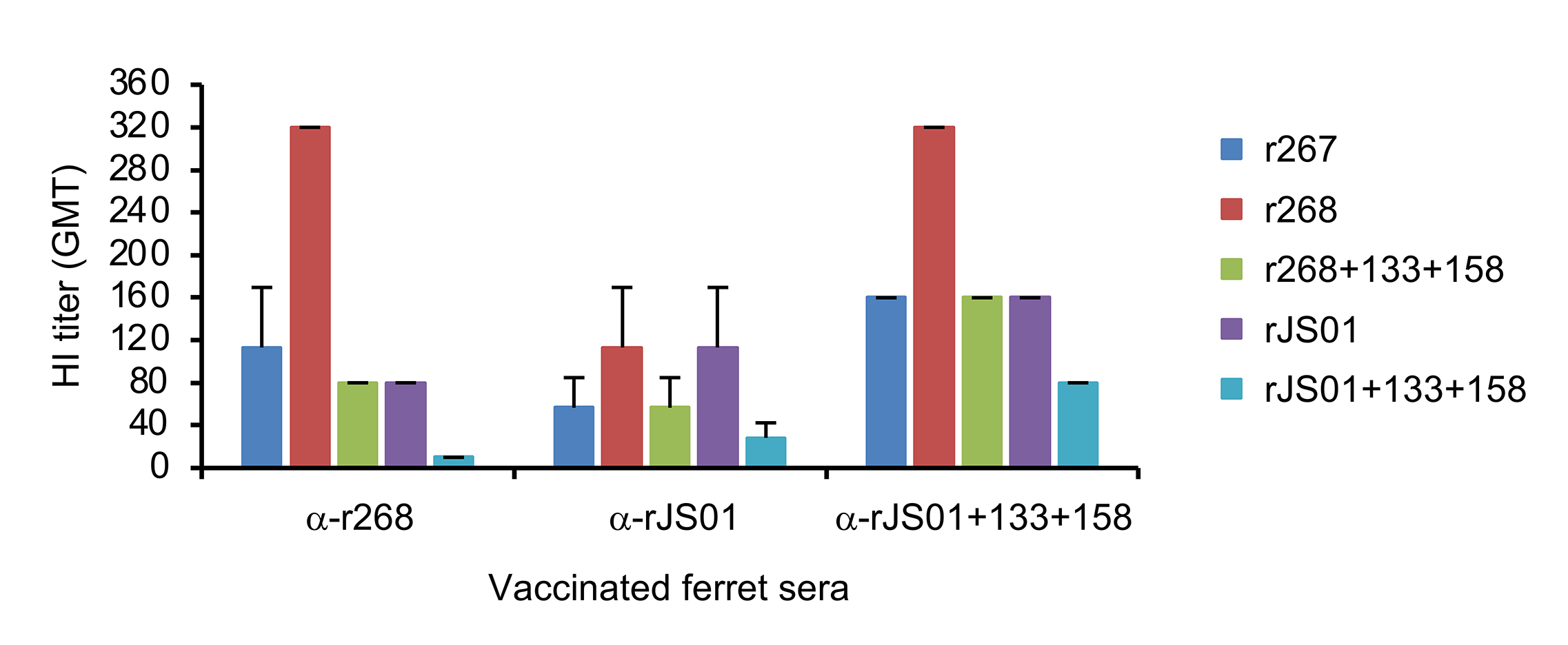

Supplement: S5 Fig — The antisera (a-r268, a-rJS01, and a-rJS01+133+158) of ferrets were used for the HI assay to examine their immunogenic profiles against H7N9 candidate vaccines (r267, r268, r268+133+158, rJS01, and rJS01+133+158). The ferrets in the nonvaccinated mice were immunized with PBS. Error bar denotes SD. Please see S7 Data for the numerical values used in S5 Fig. HI, hemagglutination inhibition; NLG, N-linked glycosylation; PBS, phosphate-buffered saline; SD, standard deviation. (TIF) [file pbio.3001024.s005.tif]

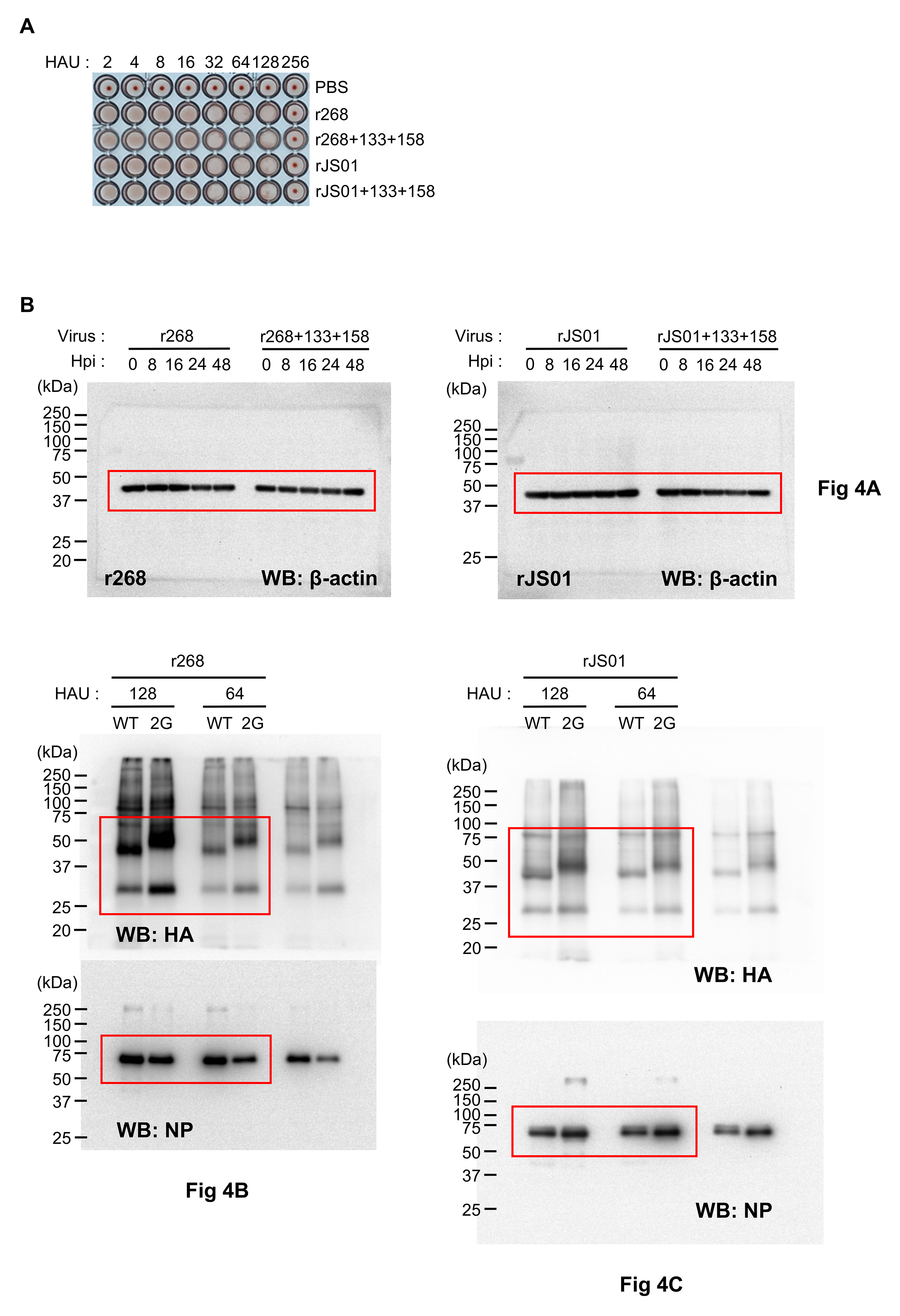

Supplement: S6 Fig — To support the results presented in Fig 4A–4C, raw data files of the HA (A) and western blot (B) assays were presented. Indications for lane and experimental conditions were also presented. Raw data of Fig 4A were obtained from the MDCK cell lysates samples and those of Fig 4B and 4C from the MDCK cell supernatants. 2G indicated 2 NLGs added at HA residues 133 and 158. HA, hemagglutinin; MDCK, Madin-Darby canine kidney; NLG, N-linked glycosylation. (TIF) [file pbio.3001024.s006.tif]

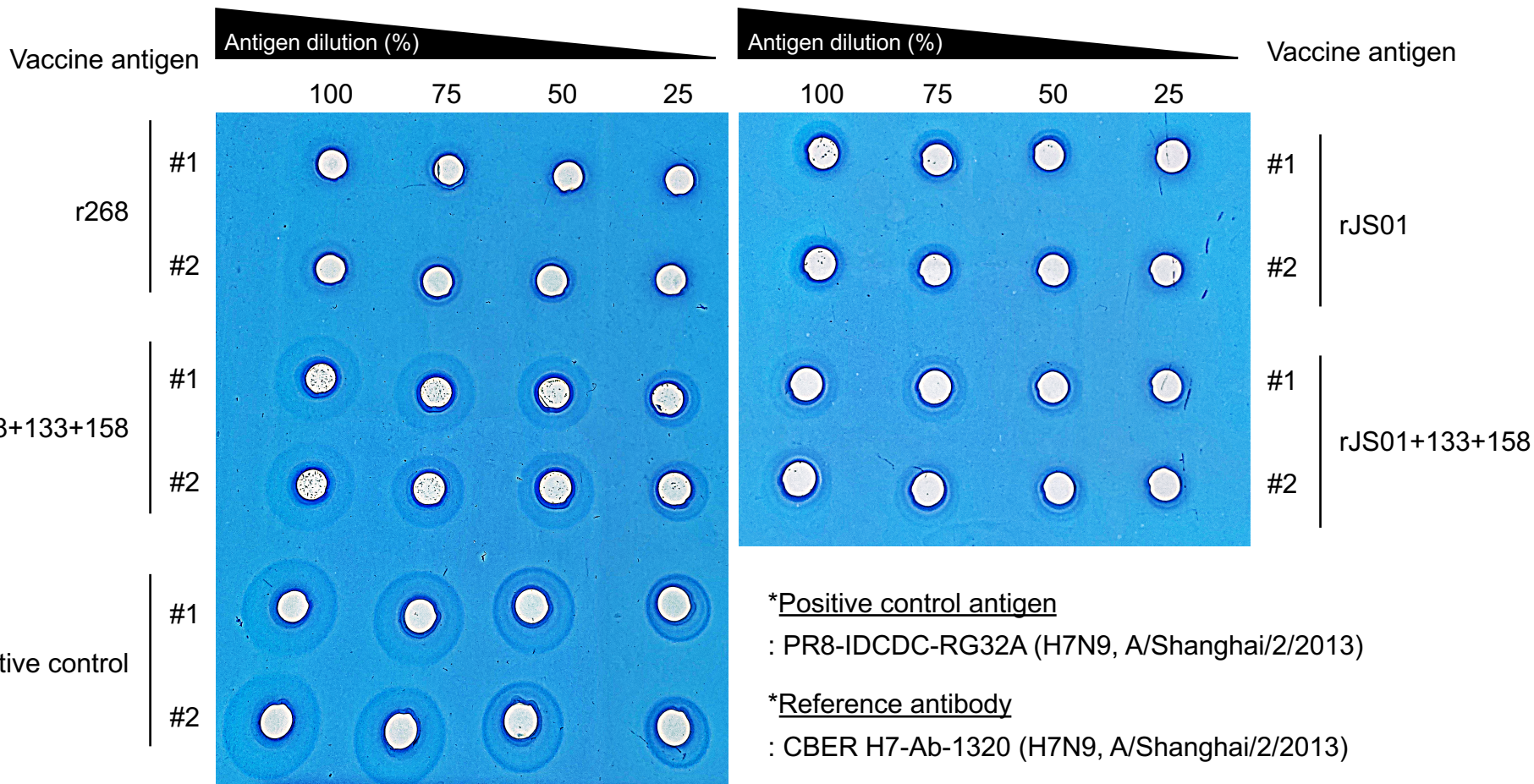

Supplement: S7 Fig — To quantify the HA protein contents in each vaccine antigen, an antigen-antibody precipitation reaction was performed in the SRID assay. PR8-IDCDC-RG32A of A/Shanghai/02/2013 was used as a positive control antigen, and all antigens were reacted with a reference antibody, CNER H7-Ab-1302 (anti-HA antibody of A/Shanghai/02/2013). The quantified HA protein contents were presented in S5 Table. (PDF) [file pbio.3001024.s007.pdf]

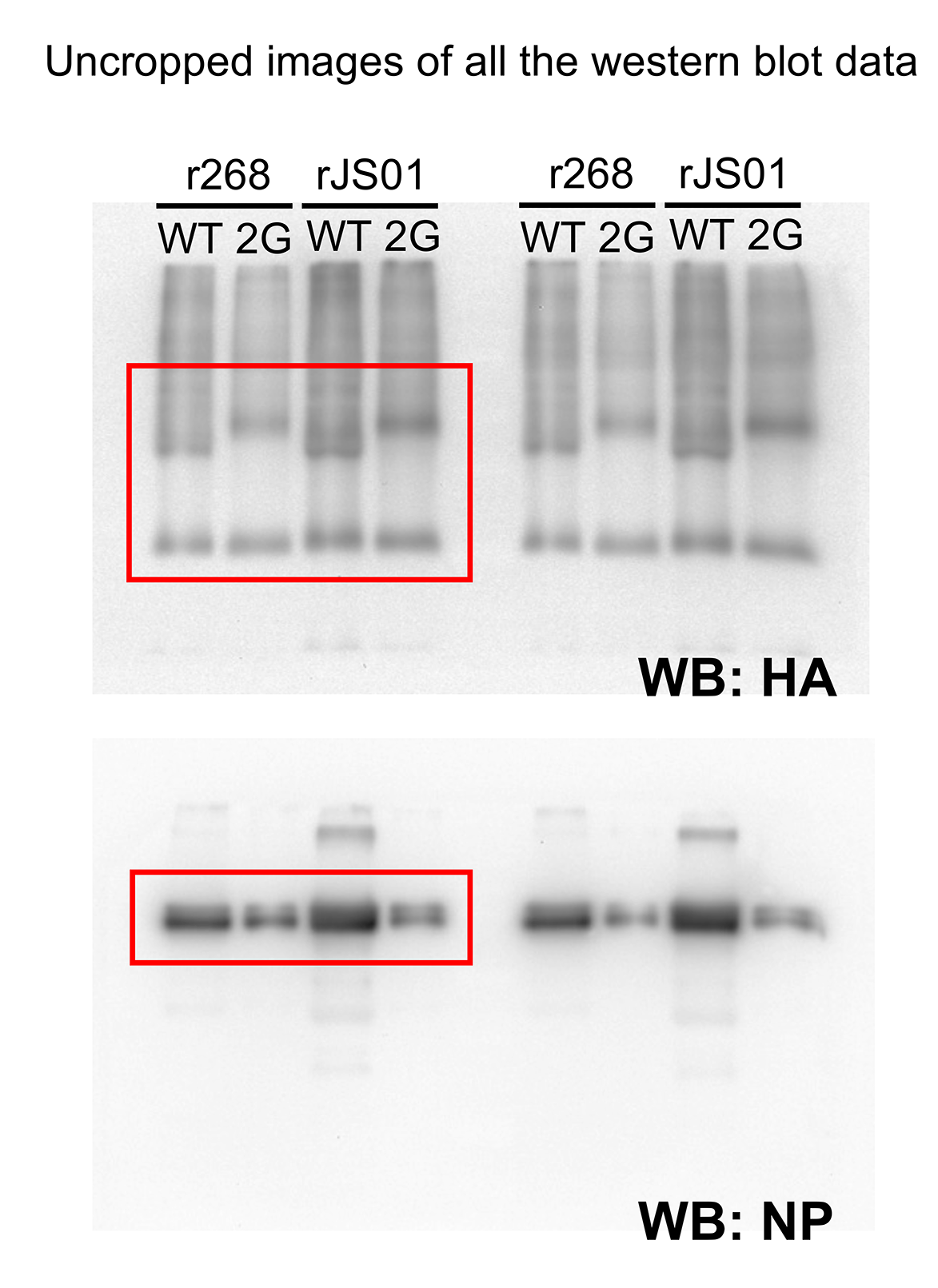

Supplement: S8 Fig — To support the results presented in Fig 4D, raw data files of the western blot assay were presented. Raw data were obtained from the quantified (1 mg concentration of the HA protein dose) vaccine antigens. 2G indicated 2 NLGs added at HA residues 133 and 158. (TIF) [file pbio.3001024.s008.tif]
