# Supplementary material for: Glycosylation generates an efficacious and immunogenic vaccine against H7N9 influenza virus
Source: PLoS Biol. 2020 Dec 23;18(12):e3001024. doi: 10.1371/journal.pbio.3001024 (PMC7757820; doi:10.1371/journal.pbio.3001024)
Supplement: S9 Fig — Specifications of a reference antigen were provided in the product circular. (PDF) [file pbio.3001024.s009.pdf]

PRODUCT CIRCULAR

INACTIVATED INFLUENZA CBER REFERENCE ANTIGEN

A/Shanghai/02/2013 (H7N9) PR8-IDCDC-RG32A

Lot: 78

For Use in Single Radial Immunodiffusion Assay

Instructions for Use:

This lyophilized reference antigen is zonally purified, formalin inactivated, whole virus preparation made from reassortant PR8-IDCDC-RG32A of A/Shanghai/02/2013 and to be used in determining the potency of inactivated Influenza A/Shanghai/02/2013 virus vaccines by Single-Radial-Immunodiffusion (SRID) assay using the CBER authorized antibodies reagent for A/Shanghai/02/2013.

Lyophilized vials should be stored at -15°C or below.

Each vial should be reconstituted in 1.0 mL of deionized water.

Ensure complete reconstitution of lyophilized antigen prior to use.

Users should determine the stability of the material according to their own methods of preparation, storage, and use.

No attempt should be made to weigh out any portion of the freeze-dried material.

A/Shanghai/02/2013 (H7N9) PR8-IDCDC-RG32A, Lot: 78, contains 60 µg HA per vial.

For additional information please contact Dr. Manju Joshi at 301-827-7917 or Manju.Joshi@fda.hhs.gov

---

Special Methods and References:

Reference Inactivated Influenza Antigen should be used according to the method described by Wood, JM; et al; Journal of Biological Standardization, 1977, 5, 237-247; Williams, MS; et al; Journal of Biological Standardization, 1980, 8, 289-296; and Williams, M.S., Veterinary Microbiology, 1993, 37, 253-262.

When preparing test dilutions according to this method, use only Zwittergent 3-14 detergent (*e.g.*, Calbiochem Behring, La Jolla, CA, USA or equivalent) as the use of other detergents may result in inaccurate potency values.

For gel analysis and comparison, CBER recommends measuring the SRID ring size from the precipitin ring's outer diameter.

This antigen is distributed by the Division of Biological Standards and Quality Control, Office of Compliance and Biologics Quality, Center for Biologics Evaluation and Research, United States Food and Drug Administration for the laboratory determination of potency or identity of inactivated influenza virus vaccines, made from A/Shanghai/02/2013 viruses.

Intended Use:

This product is NOT for Human Use and for in vitro laboratory use only. No known hazards are associated with this reagent.

Distributed by United States Food and Drug Administration's Center for Biologics Evaluation and Research (CBER) and is intended for use as a standard, reference material, or a reagent in laboratory work in relation to biological research, manufacturing, or quality control testing of biological products or in the field of in vitro diagnostics.
