# Supplementary material for: Glycosylation generates an efficacious and immunogenic vaccine against H7N9 influenza virus
Source: PLoS Biol. 2020 Dec 23;18(12):e3001024. doi: 10.1371/journal.pbio.3001024 (PMC7757820; doi:10.1371/journal.pbio.3001024)
Supplement: S10 Fig — Specifications of a reference antibody were provided in the product circular. (PDF) [file pbio.3001024.s010.pdf]

PRODUCT CIRCULAR

Antibodies to A/Shanghai/02/2013

INFLUENZA VIRUS HAEMAGGLUTININ (HA)

CBER Lot: H7-Ab-1402

For Use in Single Radial Immunodiffusion Assay

Instructions for Use:

This influenza haemagglutinin (HA) antibodies reagent CBER Lot: H7-Ab-1402 was prepared for use in Single Radial Immunodiffusion Assay for determination of HA content of A/Shanghai/02/2013 like viruses during manufacture and testing of inactivated influenza vaccines.

Lyophilized vials should be stored at 2 to 8°C or below. Each vial should be reconstituted in 2 mL of purified, distilled, or deionized water. Reconstituted material can be used for up to a week if stored at 2 to 8°C or below. Avoid freeze thaw cycles if reconstituted material is stored frozen.

Users should determine the stability of the material according to their own methods of preparation, storage, and use, if used beyond 7 days of reconstitution.

CBER recommends using 14 to 18 µL of reconstituted antibodies per mL of agarose when testing A/Shanghai/02/2013 antigens at a concentration of approximately 30 µg of haemagglutinin per mL. It may be necessary to change the antibodies concentration according to the specific methodology used in the laboratory.

For additional information please contact Dr. Manju Joshi at 301-827-7917 or Manju.Joshi@fda.hhs.gov

Special Methods and References:

Antibodies to Influenza Virus Haemagglutinin should be used according to the method described by Wood, JM; et al; Journal of Biological Standardization, 1977, 5, 237-247; Williams, MS; et al; Journal of Biological Standardization, 1980, 8, 289-296; and Williams, M.S., Veterinary Microbiology, 1993, 37, 253-262.

For gel analysis and comparison, CBER recommends measuring the SRID ring size from the outer diameter of the precipitin ring.

These antibodies were generated by immunizing and boosting sheep with bromelain-cleaved HA purified from egg grown A/Shanghai/02/2013 reassortant, PR8-IDCDC-RG32A. Additional boosting was done with influenza H7 (A/Shanghai/02/2013) virus-like particles prepared in Vero cells (1). Immunization in sheep was performed in compliance with an animal research protocol approved by the Institutional Animal Care and Use Committees of the Center of Biologics Evaluation and Research (CBER) and the NIH.

This antibody reagent was lyophilized and tested in the Division of Biological Standards and Quality Control, Office of Compliance and Biologics Quality, CBER for distribution by FDA/CBER for the laboratory determination of potency or identity of inactivated influenza virus vaccines, made from A/Shanghai/02/2013 like viruses.

1 - Schmeisser et al., (2012) Vaccine 30:3413– 3422

Intended Use:

This product is NOT for Human Use and for *in-vitro* laboratory use only. No known hazards are associated with this reagent.

Distributed by United States Food and Drug Administration's Center for Biologics Evaluation and Research (CBER) and is intended for use as a standard, reference material, or a reagent in laboratory work in relation to biological research, manufacturing, or quality control testing of biological products or in the field of *in-vitro* diagnostics.
