# Supplementary material for: Glycosylation generates an efficacious and immunogenic vaccine against H7N9 influenza virus
Source: PLoS Biol. 2020 Dec 23;18(12):e3001024. doi: 10.1371/journal.pbio.3001024 (PMC7757820; doi:10.1371/journal.pbio.3001024)
Supplement: S1 Table — (DOCX) [file pbio.3001024.s011.docx]

**S1 Table. Frequency of NLG modifications on HAs from selected H7-subtype viruses.**

|  | NLG % at a given residue^§^ (from unique H7 HA sequences, n = 1,361) | | | | | | | |
| --- | --- | --- | --- | --- | --- | --- | --- | --- |
|  | HA1 region |  |  |  |  | HA2 region |  |  |
| Subtype | 22 | 38 | 133 | 158 | 240 | 82 | 154 | No. of sequences |
| H7N1 | 99.24 (131) | 100 (132) | 30.30 (40) | 23.48 (31) | 100 (132) | 100 (132) | 99.24 (131) | 132 |
| H7N2 | 99.00 (198) | 100 (200) | 4.50 (9) | 4.50 (9) | 99.5 (199) | 100 (200) | 100 (200) | 200 |
| H7N3 | 100 (426) | 98.12 (418) | 7.75 (33) | 4.23 (18) | 100 (426) | 99.53 (424) | 100 (426) | 426 |
| H7N7 | 97.63 (247) | 100 (253) | 5.53 (14) | 1.19 (3) | 100 (253) | 100 (253) | 100 (253) | 253 |
| H7N9 | 100 (350) | 100 (350) | 1.43 (5) | 0.29 (1) | 100 (350) | 100 (350) | 100 (350) | 350 |
| Total | 99.34 (1,352) | 99.41 (1,353) | 7.42 (101) | 4.56 (62) | 99.93 (1,360) | 99.85 (1,359) | 99.93 (1,360) | 1,361 |

^§^H3 numbering.
