# Supplementary material for: Glycosylation generates an efficacious and immunogenic vaccine against H7N9 influenza virus
Source: PLoS Biol. 2020 Dec 23;18(12):e3001024. doi: 10.1371/journal.pbio.3001024 (PMC7757820; doi:10.1371/journal.pbio.3001024)
Supplement: S2 Table — (DOCX) [file pbio.3001024.s012.docx]

**S2 Table. HA NLG signatures of the mutant viruses and their viral titers.**

|  | NLG status at HA residue | | |  |
| --- | --- | --- | --- | --- |
| Reassortant virus | 133 | 158 | 240 | Titer (PFU/ml) |
| rH7 |  |  | ○ | 4.3 ✕ 10^7^ |
| rH7+133 | ○ |  | ○ | 5.7 ✕ 10^6^ |
| rH7+158 |  | ○ | ○ | 2.9 ✕ 10^7^ |
| rH7-240 |  |  |  | 4.1 ✕ 10^5^ |
| rH7+133+158 | ○ | ○ | ○ | 1.6 ✕ 10^8^ |
