# Supplementary material for: Glycosylation generates an efficacious and immunogenic vaccine against H7N9 influenza virus
Source: PLoS Biol. 2020 Dec 23;18(12):e3001024. doi: 10.1371/journal.pbio.3001024 (PMC7757820; doi:10.1371/journal.pbio.3001024)
Supplement: S3 Table — (DOCX) [file pbio.3001024.s013.docx]

**S3 Table. Pathogenicity of the HA NLG mutant viruses in BALB/c mice.**

| Virus | MID_50_ | MLD_50_^†^ | Maximum body weight loss (%)^‡^, mean ± SD^§^ (DPI^¶^) |
| --- | --- | --- | --- |
| rH7 | 10^1.7^ | >10^6.5^ | 9.6 ± 3.58 (6) |
| rH7+133 | 10^1.3^ | >10^6.5^ | No reduction in body weights |
| rH7+158 | 10^1.2^ | 10^5.5 ± 0.3^ | 24.79 (9) |
| rH7-240 | 10^1.7^ | >10^6.5^ | No reduction in body weights |
| rH7+133+158 | 10^1.9^ | >10^6.5^ | 19.97 ± 1.77 (8) |

^†^, based on the survival rates of the mice infected at 10^5^ and 10^6^ PFU titers in S3 Fig; ^‡^, based on the body weight changes of the mice infected at a 10^5^ PFU titer of each virus in S3 Fig; ^§^, Standard deviation; ^¶^, days post-infection.
