# Supplementary material for: Glycosylation generates an efficacious and immunogenic vaccine against H7N9 influenza virus
Source: PLoS Biol. 2020 Dec 23;18(12):e3001024. doi: 10.1371/journal.pbio.3001024 (PMC7757820; doi:10.1371/journal.pbio.3001024)
Supplement: S4 Table — (DOCX) [file pbio.3001024.s014.docx]

**S4 Table. Pairwise HI (PRNT) titers of guinea pig antisera.**

|  | GMT ± SD^†^ of guinea pig antisera | | | | |
| --- | --- | --- | --- | --- | --- |
| Virus | rH7 | rH7+133 | rH7+158 | rH7-240 | rH7+133+158 |
| rH7 | 320 ± 1.76 | 160 | 269.09 ± 1.41 | 134.54 ± 1.41 | 160 |
|  | (452.55 ± 1.63)^‡^ | (113.14 ± 1.63) | (452.55 ± 55) | (226.27 ± 1.63) | (80) |
| rH7+133 | 67.27 ± 2.83 | 80 ± 2.67 | 47.57 ± 1.94 | 80 | 113.14 ± 1.49 |
|  | (28.28 ± 1.63) | (113.14 ± 1.63) | (14.14 ± 1.63) | (40 ± 2.67) | (40) |
| rH7+158 | 320 ± 1.76 | 80 ± 1.76 | 269.09 ± 1.41 | 113.14 ± 1.49 | 67.27 ± 1.41 |
|  | (320) | (40) | (320) | (113.14 ± 1.63) | (40) |
| rH7-240 | ^¶^ n.d. | n.d. | n.d. | n.d. | n.d. |
|  | (905.1 ± 1.63) | (640) | (1280 ± 2.67) | (640) | (320) |
| rH7+133+158 | 40 ± 1.76 | 47.57 ± 1.94 | 28.28 ± 1.49 | 28.28 ± 1.49 | 40 |
|  | (80^§^) | (56.57 ± 1.63) | (20 ± 2.67) | (40 ± 2.67) | (28.28 ± 1.63) |

^†^GMT, geometric mean titer from four (HI assay) and two (PRNT) independent experiments; s.d., standard deviation; ^‡^PRNT titers are presented in parenthesis; ^¶^n.d., not determined; ^§^, determined in one experiment. Serum of naïve guinea pigs that was used as a control resulted in no HI (PRNT) reaction.
