# Supplementary material for: Glycosylation generates an efficacious and immunogenic vaccine against H7N9 influenza virus
Source: PLoS Biol. 2020 Dec 23;18(12):e3001024. doi: 10.1371/journal.pbio.3001024 (PMC7757820; doi:10.1371/journal.pbio.3001024)
Supplement: S5 Table — (DOCX) [file pbio.3001024.s015.docx]

**S5 Table. HA protein concentration included in the vaccine antigens.**

| Vaccine antigen | HA concentration (μg/ml) | ratio to WT |
| --- | --- | --- |
| r268 | 3.93 | 1 |
| r268+133+158 | 22.9 | 5.83 |
| rJS01 | 4.54 | 1 |
| rJS01+133+158 | 10.83 | 2.39 |
